# A step towards Balkan *Capsicum annuum* L. core collection: Phenotypic and biochemical characterization of 180 accessions for agronomic, fruit quality, and virus resistance traits

**DOI:** 10.1371/journal.pone.0237741

**Published:** 2020-08-17

**Authors:** Amol N. Nankar, Velichka Todorova, Ivanka Tringovska, Gancho Pasev, Vesela Radeva-Ivanova, Valentina Ivanova, Dimitrina Kostova

**Affiliations:** 1 Center of Plant Systems Biology and Biotechnology (CPSBB), Plovdiv, Bulgaria; 2 Maritsa Vegetable Crops Research Institute (MVCRI), Plovdiv, Bulgaria; University of Tsukuba, JAPAN

## Abstract

Region-specific local landraces represent a germplasm diversity adapted and acclimatized to local conditions, and are ideal to breed for targeted market niches while maintaining the variability of heirloom traits. A collection of 180 pepper accessions, collected from 62 diverse locations across six Balkan countries, were characterized and evaluated for phenotypic and biochemical variation during a multi-year environment. An assortment of 32 agro-morphological, fruit quality, and virus resistance traits were evaluated, and the top 10% accessions were identified. A wide range of trait variation concerning plant architecture, inflorescence and fruit traits, yield and fruit quality was observed, and appreciable variation was noticed. According to hierarchical clustering, six distinct clusters were established based on pre-defined varietal groups. Divergence among accessions for phenotypic and fruit compositional variability was analyzed, and eight principal components were identified that contributed ~71% of the variation, with fruit shape, width, wall thickness, weight, and fruit quality traits being the most discriminant. Evaluation of the response to tobacco mosaic virus (TMV) and pepper mild mottle mosaic virus (PMMoV) showed that 24 and 1 accession were resistant, respectively while no tomato spotted wilt virus (TSWV) resistance was found. Considerable diversity for agro-bio-morphological traits indicates the Balkan pepper collection as good gene sources for pre-breeding and cultivar development that are locally adapted.

## Introduction

Over the last few centuries, *Capsicum* has distributed and spread across every part of the world [1]. As it distributed and acclimatized in different regions, it has undergone concurrent selection for local adaptations [2], providing new varieties derived from the original populations. However, the phenotypic and genetic variability of these local varieties remains poorly understood in some regions [3]. *Capsicum* diversity is a reflection of different factors that are constantly evolving on both the spatial and temporal scale. These factors depend on the relationship between local varieties or forms, the socio-cultural significance associated with specific varietal types, cultivation practices, and potential gene transfer between modern cultivars and wild relatives [4].

Local heirloom varieties or landraces are maintained by farmers [5] and are passed on from generation to generation. The usefulness of local landraces is critical for crop production sustainability and the sustenance of low-input conventional farming [6–7]. Locally acclimatized and adapted landraces are ideally suited to breed for local market niches, although the size of huge germplasm collections obstructs the efficient use of genetic resources in crop improvement [8]. Hence, the creation of a core collection is essential in order to reduce the redundancy amongst landrace accessions and to select, conserve, and utilize true representatives of genetic diversity [9]. Core collections help represent germplasm diversity [10], reduce germplasm maintenance cost [11], improve varietal identification [12], efficiently utilize germplasm diversity [13], and support pre-breeding efforts [14].

The creation of a core collection and its subsequent conservation in gene banks and utilization for breeding requires germplasm collection to be properly evaluated and characterized [15–17]. Morphological evaluation of regionally adapted and acclimatized genetic resources has proven essential in core collection creation and in harnessing plant genetic diversity [18]. Phenotypic characterization can be a daunting task that needs a well-thought strategy and prolonged efforts [19]. Even then, phenotypic characterization is difficult to evaluate when inter-varietal differences are minute and subtle [20]. Conventionally, the characterization of pepper genetic resources has always been wont to investigate phenotypic variability. Innumerable studies are conducted to elucidate the phenological and agronomic variability among local pepper populations in former Yugoslavia [21], Bulgaria [22–23], South Africa [24], Italy [25], Turkey [26], Mexico [27], Brazil [28], India [29], Peru [30], Argentina [31], Bolivia [32], and Spain [33].

The Balkans have a rich tradition of growing endemic types of peppers that do not seem to be found in other parts of the world [34–35], and appear to be a “secondary region for pepper diversity” [36]. Several studies in the past have investigated Balkan pepper diversity and noticed appreciable diversity for fruit compositional [37], and morpho-colorimetric descriptors [38]. However, no study has yet been published that demonstrates comprehensive morphological characterization of a large pepper collection. This study was designed to provide such a comprehensive morphological characterization. The objectives of this study were to: a) evaluate a set of pepper accessions comprised of local varieties, local forms, landraces, and breeding lines collected from six Balkan countries, b) assess their morphometric and fruit compositional variations to create a Balkan pepper core collection and identify the best performing pepper accessions, and c) identify the resistance sources against tobamoviruses (TMV and PMMoV) and TSWV.

## Materials and methods

### Germplasm material

**Fig 1:**
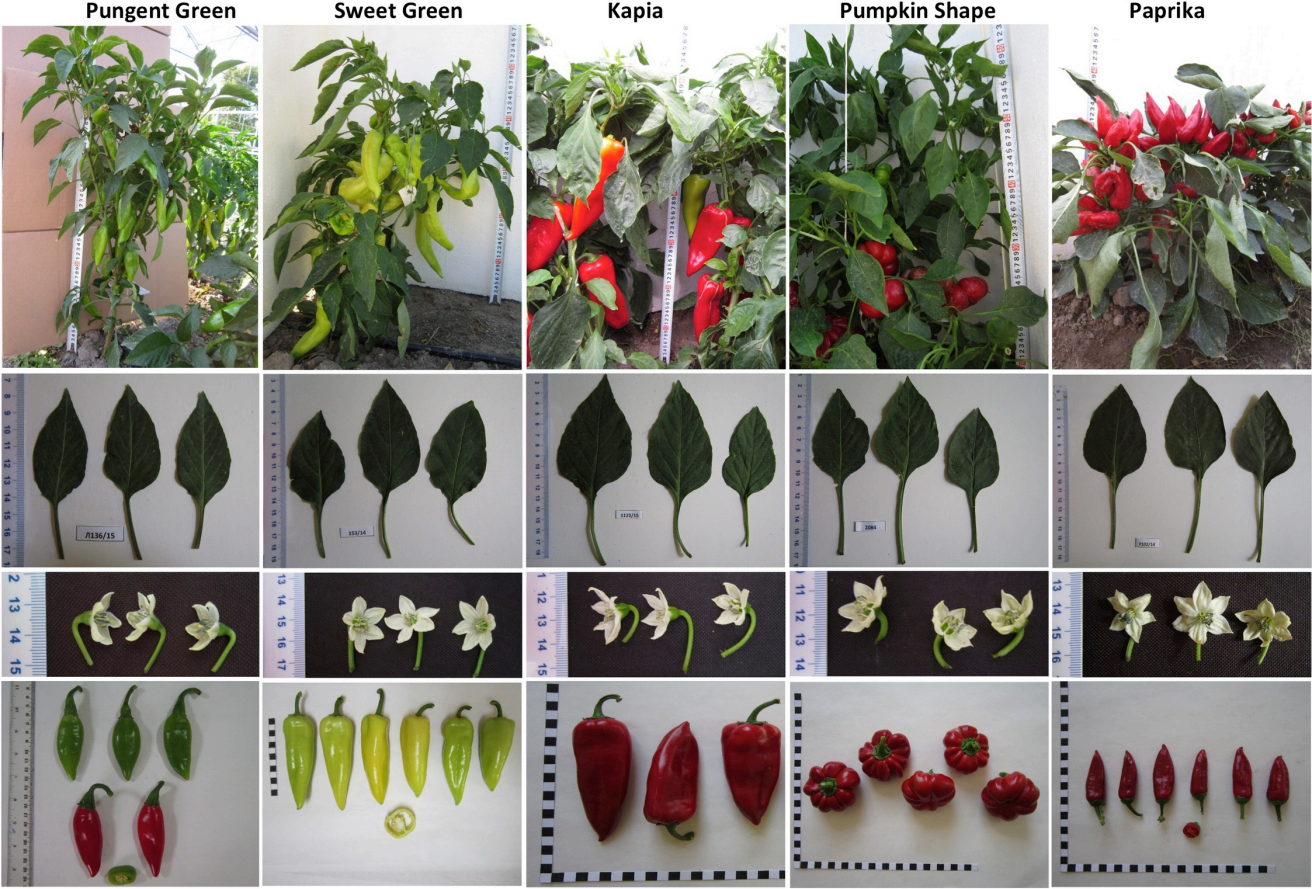
The whole plant, leaf, flower, and fruit shape diversity of accessions representing pre-defined pepper Varietal Groups (VGs). Columns from left to right represent accessions from Pungent (A), Sweet green (B), Kapia (C), Pumpkin shape (D), and Paprika (E) VGs, respectively. Whole plant, leaf, flower morphology, and fruit images are shown in the top to bottom rows, respectively.

A total of 180 *Capsicum annuum* L. accessions representing six Balkan countries (Albania, Bulgaria, Greece, Macedonia, Romania, and Serbia) were included during this investigation. Accessions originated and/or were collected from 62 geographical sites and their geographical distribution across the Balkan Peninsula is shown in a previously published high-throughput fruit phenotyping study [38]. Commonly recognized accessions in Balkan pepper growing regions, belonging to different pre-defined varietal groups (VGs) based on end-use, include Pungent, Sweet green, Kapia, Pumpkin shape, and Paprika, and these are shown in Fig 1.

### Experimental design

Each accession was represented by 30 plants during an open field plot trial with three replications in a randomized complete block design in MVCRI, Plovdiv, Bulgaria (GPS Coordinates: 42°10'35.3"N 24°45'50.5"E) during the period 2018–2019.

### Seed germination, transplanting, and plant growth

Seeds were sown at the end of March in an unheated greenhouse, and six-week-old pepper seedlings were transplanted in the middle of May in a two-rowed planting scheme (100+60/15 cm). All accessions were grown in accordance with adopted technology for mid-early field production. The fertilization, irrigation, plant protection, and microclimate were the same for all genotypes.

### Trait characterization

#### Phenotypic and morphological trait characterization

During different phenological growth stages, pre-harvest and post-harvest characterizations were mainly based on 26 conventional descriptors related to vegetative data—3 (plant growth habit, plant height, and nodal anthocyanin), inflorescence—4 (number of pedicel per axil, pedicel position at anthesis, corolla colour, and corolla spot), fruit—12 (calyx margin shape, annular construction at the junction of calyx and peduncle, fruit position, fruit colour at an immature and mature stage, fruit shape, fruit shape at pedicel attachment and blossom end, fruit size, fruit cross-sectional corrugation, fruit wall thickness, fruit set, and fruit pungency), seed—1 (seed colour), agronomic data—6 (varietal type, fruit set, flowering earliness, maturity earliness, fruit yield per plant, and use). Additionally, 10 quantitative traits were also studied including plant height (cm), stem length (cm), embranchment number at the first order, fruit length (cm), fruit width (cm), number of locules, fruit wall thickness (mm), fruit weight (g), fruit usable part (g), and productivity (kg per plant). The Evaluation of plant traits was done after the end of active vegetation growth while fruit traits were measured at the second harvest. Morphological traits were assessed on two randomized plants and fruits for each of the three replications, while productivity was measured on one plant for each of the three replications.

#### Fruit compositional quality

Fruits were collected before maturity (Pungent and Sweet green) and at full maturity (Kapia, Pumpkin shape, and Paprika). Collected fruits were characterized by the following quality traits: total soluble solids (TSS) or Brix content (%), and ascorbic acid or vitamin C (Vit C) content (mg/100g FW) from the freshly homogenized sample. TSS was determined by a hand-held refractometer and Vit C was determined by Tillman’s reaction [39]. Total polyphenols (TP), and antioxidant activity by ferric-reducing antioxidant power (FRAP) were estimated in lyophilized material. The TP (mg GAE/100g FW), and FRAP (μmolFe^2+^/g FW) extraction procedures were performed as per the optimized method described by Atanasova et al [40]. TP was quantified according to the Singleton and Rossi [41] method. FRAP was measured following the procedure originally described by Benzie and Strain [42].

#### Virus resistance evaluation

*Virus screening against TMV*, *PMMoV*, *and TSWV*. Plants were grown in trays with a peat-perlite mixture in a growth chamber at 22 ºC—25 ºC and a 14hr/10hr light/dark photoperiod during 2018. TMV (P0), PMMoV P8 strain (P1.2), and a local isolate of TSWV were used for the preparation of inoculum. Three viruses were propagated on *Nicotiana tabacum* cv. Plovdiv, *N*. *benthamiana* and *N*. *tabacum* cv. Nevrokop, respectively. Symptomatic leaves were ground in a buffer (1% K_2_HPO_4_ and 0.1% Na_2_SO_3_ pH9) at 1:10 w/v ratio and a small amount of carborundum was added as abrasive. Inoculation procedures were done by mechanical scrubbing of one leaf per plant at 4–6 leaf stage.

Two sets of 10 plants from each accession were used for inoculations with two viruses (TMV and TSWV). A detached leaf test (DLT) for PMMoV was applied on plants separated for TMV screening. From each plant, the first true leaf was cut, inoculated with PMMoV, and incubated in a humid chamber for 72–96 hrs at 25 ºC before scoring. Inoculations with TMV and TSWV were done on the remaining intact plants. Resistance to the three viruses is based on a hypersensitive reaction expressed by local lesions developed after 3 to 4 days post inoculation without systemic spread of the virus. Development of systemic symptoms, such as vein clearing on upper leaves, mosaic, and leaf deformation, is considered as a susceptible reaction to the virus.

*Molecular analysis*. Pepper accessions showing resistance to both TMV and PMMoV were evaluated for the presence of the *L*_*3*_ or *L*_*4*_ resistance allele which controls a broader spectrum of resistance to viral strains compared to alleles *L*_*1*_ or *L*_*2*_. Total genomic DNA was extracted from upper young leaves of pepper seedlings using sbeadex kit (LGC genomics). Genotyping was carried out by codominant markers PMFR11 [43] and 087H3T7 [44]. Polymerase chain reaction (PCR) amplifications were conducted in Biorad T100 (Biorad Laboratories) thermocycler in a final volume of 25 μl. The reaction mixture consisted of 1x reaction buffer, 1.5 mM MgCl_2_, μL 0.3 mM dNTPs mix, 0.4 μM of each primer, 1 U BIOTAQ™ (Bioline Reagents Ltd., London, UK), and 50 ng genomic DNA as a template. PCR temperature profile executed on the thermocycler started with an initial denaturation step at 95 ºC followed by 35 cycles of 94 ºC for 30 sec, 55 ºC for 30 sec, and 72 ºC for 1 min. A final extension for 7 min at 72 ºC was added. Endonuclease treatment of 440 bp fragment obtained with 087H3T7 primers was performed with FastDigest SspI (Thermo Fisher Scientific Inc.) in a volume of 15 μl according to manufacturers recommendations. PCR and digestion products were separated on 2% gel electrophoresis and stained by SimplySafe™ (EURx Ltd., Poland) for visualisation.

### Statistical analyses

Agro-morphological field data and fruit compositional quality data were analyzed using SAS, and R programs. The manuscript structure was inspired by previous work of biomorphological pepper diversity [45], bean phenotypic collection [19], and tomato fruit diversity [46].

#### Analysis of Variance (ANOVA)

The generalized linear model (GLM) was used to analyze quantitative traits through the F test in the ANOVA to detect significant differences using SAS software. Differences among accessions across pre-defined VGs, and within each VG were detected by Least Significant Difference (LSD) at α = 0.05.

#### Hierarchical cluster analysis

A total of 32 traits associated with plant architecture, fruit form, and fruit quality was used to establish the distinct clusters using Ward’s coefficient by agglomerative hierarchical clustering in R program using *dendextend* [47], and circular implementation of dendrogram was done using *circlize* R package [48].

#### Multivariate analysis

Multivariate analysis was utilized to comprehend the variation between accessions belonging to different end-use varietal groups. PCA parameters of eigenvalue, percent variance of different components, and accession by variable biplot were estimated by ggplot2, missMDA, FactoMineR, and Factoextra R packages. Correlation coefficient and correlation network were also constructed to understand how agro-morphological and fruit quality traits contribute to phenotypic and biochemical diversity. Correlation coefficient matrix and correlation network were constructed using *cor* function and *qgraph*, respectively.

## Results

### Agronomical and fruit quality trait characterization and between accessions variation

**Fig 2:**
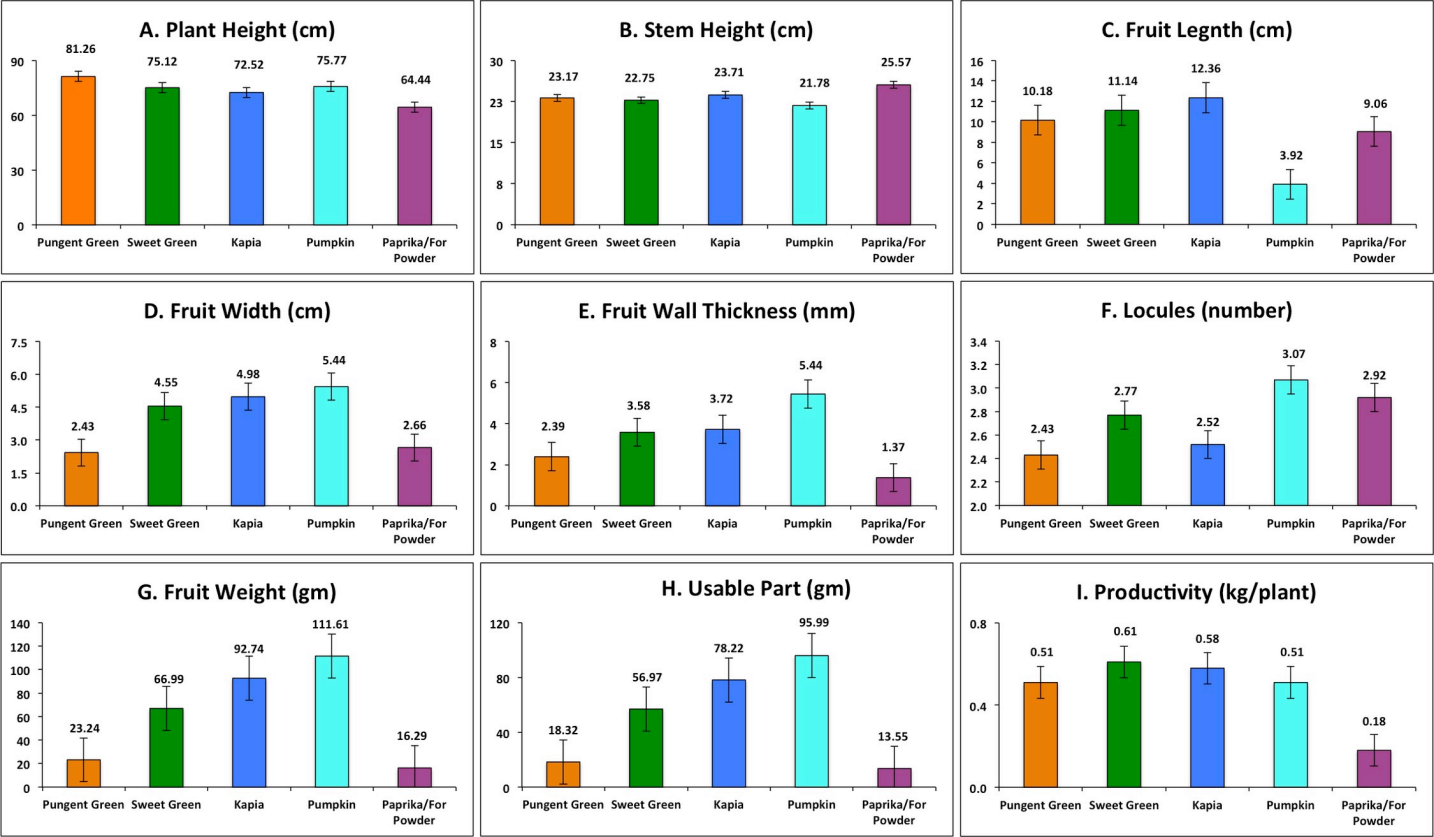
Average values of agronomical and fruit form traits for each pepper varietal group.

The investigated pepper collection comprised accessions from the Balkan region: Bulgaria (114), Serbia (28), Macedonia (16), Romania (9), Albania (7), Greece (3), and unknown origin (3). Most accessions were local forms and landraces (49%) followed by cultivars (32%), unknown population type (17%), and breeding lines (2%). Agro-morphological trait variation between varietal groups (VGs) evaluated across both years as well as separately for 2018, and 2019 is shown in Table 1A whereas between VGs variation across both years and separately for 2018 and 2019 is shown in S1A, S2A, and S3A Tables, respectively. Across years and within 2018 and 2019 productivity, usable part, fruit weight, fruit length, and stem height had greater variation compared to other traits while fruit width displayed the least variation followed by plant height (Table 1A). Mean, maximum, and minimum values for 10 agro-morphological characters revealed the presence of a broad range of phenotypic variation in the studied Balkan pepper collection (Table 1A). The individual accessions means for agro-morphological traits evaluated during both years is shown in S4 Table. Across all VGs, intermediate plant height was dominant for 90% of tested accessions, whereas 8% were tall (over 100 cm) and largely found in Pungent green VG while most short (below 50 cm) accessions were seen in Paprika (S1 Table and Fig 2A). Highest stem height was measured (25.57 cm) in Paprika and least (21.78 cm) in Pumpkin shape (S1 Table and Fig 2B). Noticeable variation in fruit form was observed between longest and shortest fruits reported from Kapia and Pumpkin VGs, respectively (Fig 2C) and between widest and thinner fruit observed in Pumpkin and Paprika VGs, respectively (S1 Table and Fig 2D). Fruit wall thickness, fruit weight, and the usable part of fruit were directly proportional with fruit width of Pumpkin and Kapia types having thicker and heavier (Table 1A) fruits while Paprika genotypes were thinner and lighter (Fig 2E and 2G). A dominant part of the collection (73.89%) showed medium productivity with over 40% of accessions having productivity above 0.60 kg/plant with mean of 0.55 kg/plant for whole collection (Table 1A). The highest productivity was reported in Sweet green (0.61 kg/plant) and least in Paprika (0.18 kg/plant) as shown in S1 Table. However, CAPS-70 (1.26 kg/plant) from Pungent group was reported as the most productive followed by CAPS-43 (1.05 kg/plant) and CAPS-74 (0.95 kg/plant) from Kapia and Pungent, respectively (Fig 2I).

**Table 1:** Descriptive stat and Analysis of Variance (ANOVA) of agro-morphological and productivity traits between accessions evaluated during across years, 2018, and 2019.

			1A. Descriptive Stat					1B. ANOVA	
Years	Trait (Unit)	N	Min	Mean	Max	CV (%)	LSD_0.05_	Accession	Accession*Year (A*Y) Inter
**1.1 Across Years**	**Plant Height** (cm)	180	32.50	75.76	114.08	13.67	8.19	15.02***	4.22***
	**Stem height** (cm)	180	10.08	23.10	31.5	21.64	3.95	7.40***	6.35***
	**Embranchment**	180	1.17	2.67	4.54	18.41	0.39	5.36***	3.31***
	**Fruit Length** (cm)	180	1.37	10.25	22.75	21.54	1.75	53.79***	2.25***
	**Fruit Width** (cm)	180	0.94	4.35	8.65	13.41	0.46	125.72***	5.96***
	**Fruit Wall Thickness** (mm)	180	1.03	3.45	6.66	19.94	0.55	38.35***	3.28***
	**Locules**	180	2.00	2.65	3.92	19.34	0.41	7.93***	2.44***
	**Fruit Weight** (g)	180	1.23	66.62	180.64	24.40	12.85	84.08***	4.94***
	**Usable Part** (g)	180	0.84	56.20	160.58	26.44	11.75	78.49***	5.39***
	**Productivity** (kg/plant)	180	0.11	0.55	1.26	33.90	0.21	5.50***	2.64***
**1.2. 2018**	**Plant Height** (cm)	180	30.00	77.53	123.33	12.92	11.21	11.23	
	**Stem height** (cm)	180	11.00	23.97	36.67	21.07	5.65	8.58***	
	**Embranchment**	180	2.00	2.62	5.92	20.10	0.59	5.39***	
	**Fruit Length** (cm)	180	1.47	10.07	22.83	16.84	1.90	46.20***	
	**Fruit Width** (cm)	180	1.02	4.10	8.65	14.95	0.69	4.10***	
	**Fruit Wall Thickness** (mm)	180	0.92	3.34	7.44	20.91	0.78	22.35***	
	**Locules**	180	2.00	2.67	4.67	19.49	0.58	4.68***	
	**Fruit Weight** (g)	180	1.32	67.29	188.58	24.91	18.76	45.94***	
	**Usable Part** (g)	180	0.88	57.53	166.92	26.61	17.13	44.38***	
	**Productivity** (kg/plant)	180	0.08	0.56	1.39	38.12	0.34	3.61***	
**1.2. 2019**	**Plant Height** (cm)	180	35.00	73.99	104.83	14.34	11.88	8.32***	
	**Stem height** (cm)	180	7.33	22.23	33.33	22.25	5.54	5.10***	
	**Embranchment**	180	0.33	2.72	3.83	16.68	0.51	2.92***	
	**Fruit Length** (cm)	180	1.27	10.42	24.33	25.14	2.93	20.40***	
	**Fruit Width** (cm)	180	0.87	4.60	9.08	12.01	0.62	83.46***	
	**Fruit Wall Thickness** (mm)	180	1.12	3.57	6.25	19.03	0.76	19.18***	
	**Locules**	180	2.00	2.63	4.00	19.19	0.56	5.72***	
	**Fruit Weight** (g)	180	1.07	65.96	195.17	23.87	17.62	42.82***	
	**Usable Part** (g)	180	0.53	54.86	170.75	26.27	16.13	39.11***	
	**Productivity** (kg/plant)	180	0.13	0.54	1.16	28.30	0.24	5.06***	

SD: Standard Deviation; CV: Coefficient of Variation; LSD: Least Significant Differences.

* ** *** showed differences at 0.05, 0.01 and 0.001 significance level, respectively.

The genotypic fixed effects and random year effects were also tested (Table 1B) where accessions discerned highly significant differences for all morphological and productivity traits. Interactions between accession and year showed highly significant differences across all VGs except for productivity from Pumpkin shape and Paprika group (S1 Table). Highly significant F-values to test the variation among accessions suggest that variability between replicates within accession was relatively small by comparison. Since A*Y interactions were significantly different, we also tested the genotypic effects for between and within VGs variation for 2018 (S2 Table), and 2019 (S3 Table). Accessions discerned significant differences for all 10 morphological traits regardless of VGs evaluated during 2018 (S2 Table) and 2019 (S3 Table) except for non significant differences were observed for productivity in Pumpkin shape and Paprika VGs during 2018 (S2 Table) whereas non significant differences observed for embranchment in Kapia and Pumpkin shape VGs during 2019 (S3 Table).

**Fig 3:**
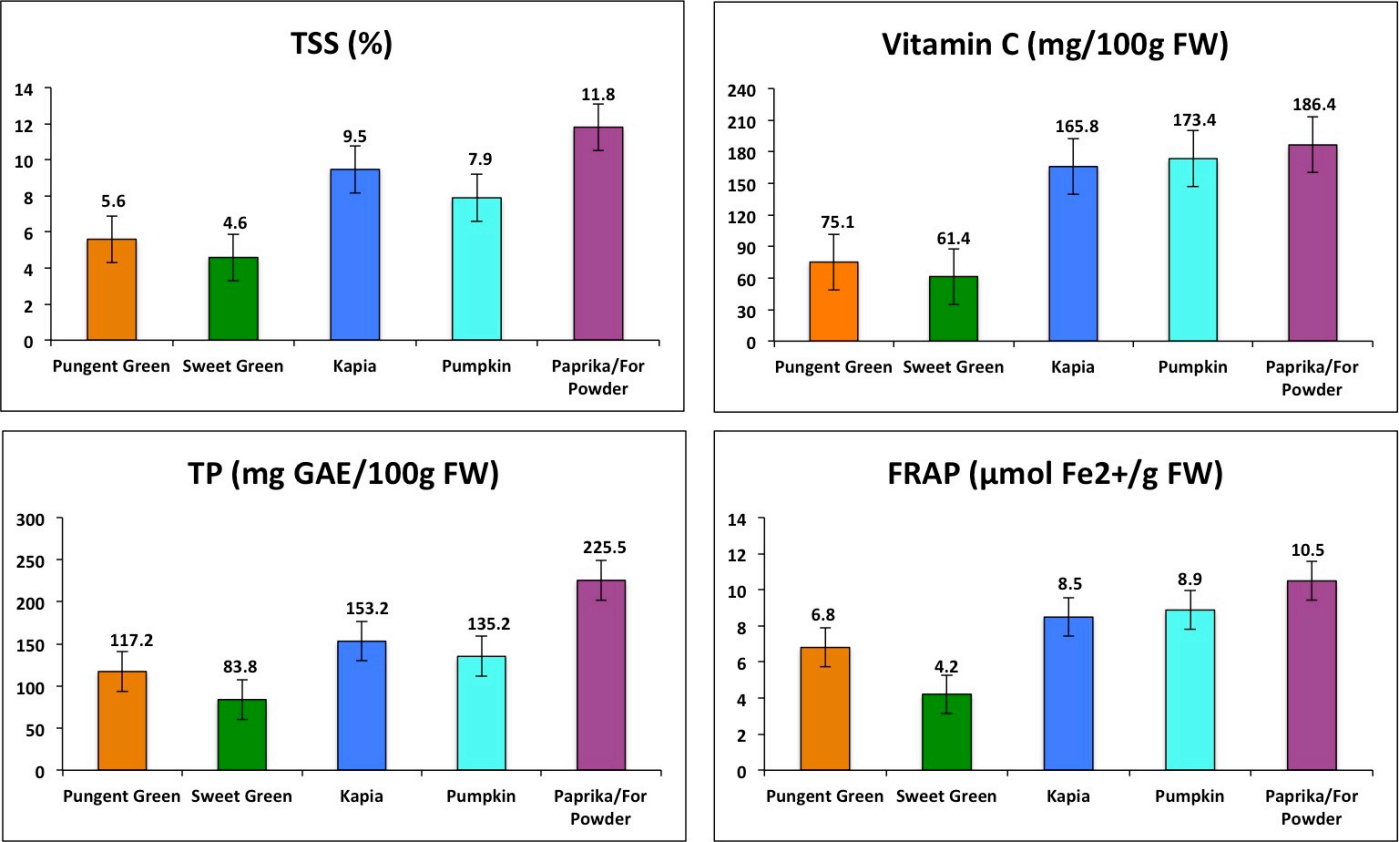
Average values of brix, vitamin C, total polyphenols and FRAP for each pepper varietal group.

Fruit compositional and quality traits were analyzed among and within VGs, and their variation is shown in Table 2. The maximum values of all tested compositional traits were observed in the Paprika while the least were in Sweet Green. Across VGs, all fruit compositional traits discerned a large range of variation for TSS (3.33–40.77), Vit C content (4.77–273.47), total phenols (48.87–414.78), and FRAP (1.58–41.56) as shown in Table 2A, and highly significant differences were observed among accessions (Table 2B). Among VGs, Paprika consistently displayed the highest mean values of TSS, Vit C, total polyphenols, and FRAP while Sweet green displayed the least (Fig 3) and difference between lowest and highest was ~2.5 fold or more. Within varietal groups, Pungent, Kapia, Pumpkin shape, and Paprika VGs had a narrow range of variation for TSS and FRAP content whereas Sweet green showed a broad range of variation. Variation for Vit C was highly variable within respective VGs except for the Paprika group (Table 2A) while accessions were significantly different for Vit C in all groups except for Pungent and Kapia type (Table 2B). The level of total phenols was highly variable in Pungent, Sweet green, and Kapia type while it was the least in Pumpkin shape and Paprika type (Table 2A); accessions were significantly different in Pungent, Sweet green, and Pumpkin shape only (Table 2B). Several pepper accessions belonging to Kapia type genotypes (CAPS-138, CAPS-133A, CAPS-173) combined the highest TSS with the highest content of total phenols and FRAP. Another two accessions, CAPS-135A (Pumpkin) and CAPS-64 (Paprika), may be distinguished by their high values of FRAP and high polyphenolics content, respectively (S5 Table). The highest Vit C was observed in several accessions harvested at full maturity stage, including CAPS-58, CAPS-56 from Kapia, CAPS-36 from Pumpkin shape, and CAPS-62 from Paprika (S5 Table). Only total phenols from Pungent group and Vit C in Pumpkin shape showed significant differences, but were otherwise nonsignificant for other traits (Table 2B). The Fig 3 shows that Paprika group has the highest mean values for all four tested compositional traits followed by Kapia and Pumpkin groups and the least in Sweet green. However, within each group, there were accessions with outstanding levels of the tested compositional traits.

**Table 2:** Descriptive statistics and Analysis of Variance (ANOVA) of fruit quality traits.

		2A. Descriptive Stat						2B. ANOVA
Varietal Groups (VGs)	Trait (Unit)	N	Min	Mean	Max	CV (%)	LSD_0.05_	Accession (F-Value)
**Across VGs**	**TSS** (%)	180	3.33	7.24	40.77	46.11	5.52	3.59***
	**Vit C** (mg/100g FW)	180	4.77	115.59	273.47	61.12	116.62	2.70***
	**TP** (mg GAE/100g FW)	180	48.87	125.28	414.78	51.06	105.59	1.84*
	**FRAP** (μmolFe2+/g FW)	180	1.58	7.27	41.46	84.23	10.10	1.70*
**Pungent**	**TSS**	50	3.67	5.72	9.27	15.34	1.75	7.17**
	**Vit C**	50	9.27	76.31	180.0	78.13	118.51	2.13
	**TP**	50	52.83	116.96	195.03	16.47	38.29	11.66**
	**FRAP**	50	1.58	6.74	15.86	11.76	1.58	49.79***
**Sweet Green**	**TSS**	48	3.33	5.62	40.77	170.35	15.92	0.98
	**Vit C**	48	4.77	66.74	273.47	53.83	59.77	8.78***
	**TP**	48	48.87	88.63	277.34	15.0	22.12	24.79***
	**FRAP**	48	1.72	5.41	41.46	175.74	15.82	1.27
**Kapia**	**TSS**	53	4.20	9.19	18.07	27.77	6.60	2.37
	**Vit C**	53	32.40	161.73	260.80	33.45	139.92	1.15
	**TP**	53	64.86	149.45	414.78	39.69	153.39	2.50
	**FRAP**	53	3.55	8.18	26.85	31.30	6.62	8.17*
**Pumpkin Shape**	**TSS**	23	6.77	7.89	9.60	11.12	1.44	2.28**
	**Vit C**	23	124.57	173.79	236.27	7.69	21.93	14.72***
	**TP**	23	111.67	135.19	175.41	13.08	29.09	3.15***
	**FRAP**	23	4.66	8.88	20.20	29.72	4.34	6.58***
**Paprika/For Powder**	**TSS**	6	10.43	11.81	13.30	11.56	2.48	1.94
	**Vit C**	6	152.80	186.44	249.60	12.48	43.32	9.13***
	**TP**	6	192.49	225.45	295.84	20.37	83.55	1.91
	**FRAP**	6	8.73	10.49	14.99	15.15	2.89	6.62**

SD: standard deviation; CV: Coefficient of Variation; LSD: Least Significant Differences.

* ** *** showed differences at 0.05, 0.01 and 0.001 significance level, respectively.

### Phenotyping of vegetative and reproductive traits

Morphological traits were phenotyped at different growth stages and characterized using 28 traits related to vegetative and reproductive variations. Characterized morphological descriptors displayed a broad range of phenotypic variation among the evaluated accessions (S1A–S1U Fig and Table 1A).

#### Vegetative traits

The accessions with erect plant growth habit dominated (62.8%) compared to accessions with compact, and prostrate (S1A Fig); however, 67 accessions belonging to the latter two habits showed 59.3% variation (Table 3). Most accessions were of intermediate plant height (S1B Fig), while short and tall accessions showed a 10.4% variation. Nodal anthocyanin on leaves (S1C Fig) was mostly varied from purple to dark purple (~81.7%).

**Table 3:** Phenotypic variation of representative plant, flower, and fruit descriptors and percentage representation in the population. The phenotypic variation analysis is inspired from the earlier work of Nankar et al [46] on tomato phenotypic diversity.

Category			Sub-category	Accessions	Accessions showing variation	% Variation
**I**	**Architecture**	**1. Plant Growth Habit**	Prostrate	3	67	59.3
			Compact	64		
			Erect	113		
		**2. Plant Height**	Short	3	17	10.4
			Intermediate	163		
			Tall	14		
**II**	**Leaf**	**3. Nodal Anthocyanin**	Green	2	96	114.3
			Light Purple	31		
			Purple	84		
			Dark Purple	63		
**III**	**Inflorescence**	**4. Flowers per Axil**	1	178	2	1.1
			2–3	1		
			2–4	1		
		**5. Pedicel Position at Anthesis**	Pendant	37	82	83.7
			Intermediate	45		
			Erect	98		
		**6. Calyx Margin Shape**	Smooth	1	51	39.5
			Intermediate	129		
			Dentate	50		
**IV**	**Fruit**	**7. Fruit Position**	Pendant	139	44	32.4
			Intermediate	11		
			Erect	30		
		**8. Immature Fruit Color**	Green	147	33	22.4
			Yellow	10		
			Red	0		
			Yellow-Green	23		
		**9. Mature Fruit Color**	Green	0	15	9.1
			Yellow	2		
			Orange	8		
			Orange-Red	1		
			Red	165		
			Brown	2		
			Yellow-Orange	2		
		**10. Fruit Size**	Very Small	4	63	53.8
			Small	12		
			Medium	44		
			Big	117		
			Very Big	3		
		**11. Fruit Set**	Low	143	37	25.9
			Intermediate	26		
			High	7		
			Very High	4		
		**12. Fruit Shape**	Elongate	35	84	91.5
			Round	7		
			Conical	96		
			Bell/Blocky	17		
			Ratundum	25		
		**13. Fruit Shape at Ped Attach**	Acute	5	104	136.8
			Obtuse	55		
			Truncate	82		
			Cordate	32		
			Lobate	6		
		**14. Fruit Shape at Blosso End**	Pointed	118	63	53.8
			Blunt	25		
			Sunken	37		
		**15. Fruit Pungency**	Non-pungent	125	55	44.0
			Low	12		
			Intermediate	39		
			High	4		
		**16. Fruit Cross-Section**	Smooth	51	103	133.7
			Slightly Corru	77		
			Intermediate	32		
			Corrugated	20		
		**17. Fruit Wall Thickness**	Very Thin	2	82	83.7
			Thin	17		
			Medium	98		
			Thick	59		
			Very Thick	4		
		**18. Fruit Yield**	Very Low	4	47	35.3
			Low	26		
			Medium	133		
			High	16		
			Very High	1		
**V**	**Earliness**	**19. Flowering**	Early	27	46	34.3
			Medium	134		
			Late	19		
		**20. Maturity**	Early	99	81	81.8
			Medium	75		
			Late	6		

#### Inflorescence traits

At anthesis, the pedicel position (S1E Fig) and calyx margin shape (S1F Fig) were erect (54.4%) and intermediate (71.7%), respectively. Around 75% accessions were of medium flowering (S1S Fig), while 34.3% variation was seen among early and late flowering accessions (Table 3). Early flowering was predominantly seen in the Pungent group while late flowering accessions were spread across varietal groups.

#### Fruit color and size

Most accessions (81.7%) showed green fruits when the fruits were immature (S1H Fig) whereas ones with yellow and yellow-green fruits discerned 22.4% variation. At the mature stage, fruits transitioned to a range of colors with the majority of accessions having red fruits (S1I Fig). A total of 15 accessions with fruit colors ranging from yellow, orange, yellow-orange, orange-red, or brown exhibited 9.1% variation (Table 3). Fruit position in 77.2% accessions (S1G Fig) was mostly pendant while 44 accessions with intermediate to erect fruit position discerned 32.4% variation. Significant fruit size variation was observed with 2/3^rd^ accessions having big fruits (S1N Fig) while accessions with variable sizes of very small to medium fruits discerned 53.8% variation (Table 3). Fruit weight displayed a broad range of 1.23g (CAPS-3) to 180.64g (CAPS-157). Fruit setting was largely low among 79.4% accessions while 1/5^th^ of accessions showing 25.9% variation for intermediate to very high fruit setting (S1R Fig).

#### Traits related to fruit shape

Fruit texture, as measured by fruit corrugation was mostly slightly corrugated (S1O Fig) and truncate and pointed shape at the peduncle attachment and blossom end (S1K and S1L Fig), respectively. The conical shape accessions represented 53.3% (S1J Fig) followed by elongate (19.4%) and Pumpkin shape (13.9%) while round and bell/blocky shape accessions represented ~12% of the total collection. Fruit wall thickness (S1P Fig) was largely varied from medium to thick (54.4% and 32.8%).

#### Yield components

Fruit yield per plant and productivity allowed us to find the yield potential of evaluated accessions. More than 70% of accessions were moderately yielding (S1U Fig) and ~10% high yielding (Table 3). Accessions with medium to high yield could be useful breeding sources in the development of high yield breeding lines.

### Virus screening for TMV, PMMoV, and TSWV resistance

Screening of 176 accessions by direct inoculation revealed that the majority of the accessions were susceptible to TMV and PMMoV and all were susceptible to TSWV. The susceptible accessions developed chlorosis and/or mosaic symptoms of TMV (S2A–S2D Fig) and PMMoV (S2E Fig) whereas development of ring chlorotic spots and mosaic is commonly caused by TSWV (S2F Fig). In TMV, the presence of necrotic local lesions (S2A Fig), net like chlorosis (S2B Fig), leaf abscission (S2C Fig), and vein clearing and chlorosis are visibly noticed during TMV occurance while necrotic lesions are commonly observed in PMMoV (S2E Fig). Resistance to TMV was only found in 23 accessions that expressed hypersensitive reaction as local lesions or vein necrosis on inoculated leaves suggested the presence of the *L*_*1*_ allele (Table 4). Two accessions from Bulgaria and one from Albania appeared to be heterogenic according to the virus resistance as their plants segregated into susceptible and resistant. The distribution of TMV resistant accessions among countries was 12, 3, 4, 3, and 1 from Bulgaria, Macedonia, Romania, Serbia, and Albania, respectively. From the entire collection only one Romanian accession (CAPS-80) was observed to be resistant to both tobamoviruses (Table 4). Both intact and DLT tests showed local lesions indicating the *L*_*3*_ or *L*_*4*_ resistance allele. The phenotypic response of all accessions to the respective viruses is shown in the S6 Table.

**Table 4:** Balkan pepper collection virus screening.

Country	Accessions	TMV		PMMoV		TSWV	
		**R**	**S**	**R**	**S**	**R**	**S**
**Bulgaria**	112	12	100	0	112	0	112
**Macedonia**	16	3	13	0	16	0	16
**Romania**	9	4	5	1	8	0	9
**Serbia**	25	3	22	0	25	0	25
**Albania**	8	1	7	0	8	0	8
**Greece**	3	0	3	0	3	0	3
**Unknown**	3	0	3	0	3	0	3
**Total**	176	23	153	1	175	0	176

TMV: Tomato Mosaic Virus; PMMoV: Pepper Mild Mosaic Virus; TSWV: Tomato Spotted Wilt Virus; R: Resistance; S: Susceptibility

**Fig 4:**
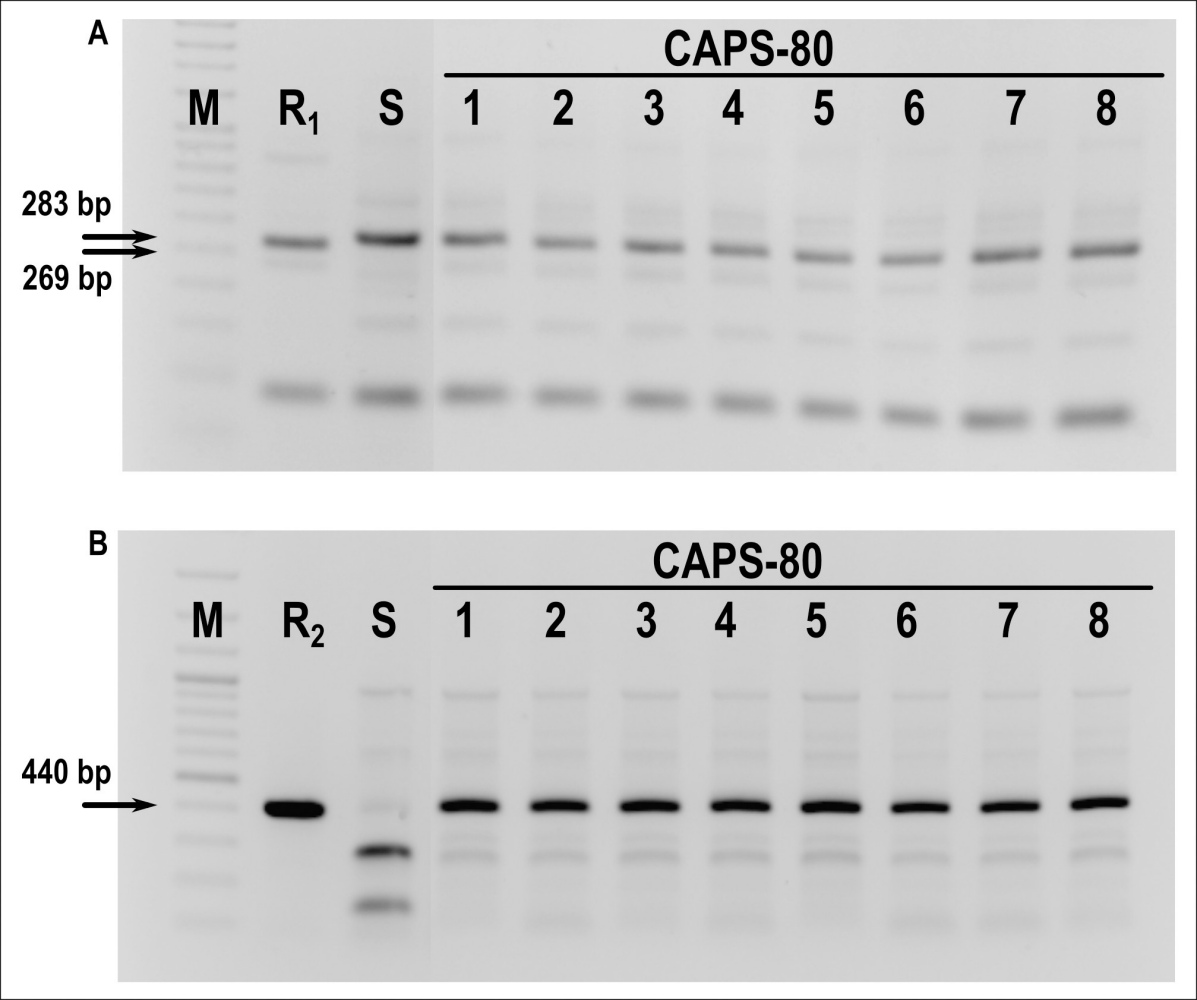
Identification of alleles for resistance to PMMoV in CAPS-80 *C*. *annuum* L. accession. A). PCR with SCAR marker PMFR11 shows absence of *L*_*3*_ allele. All individuals amplified susceptible 283bp allele. B). Restriction profile of 440bp 087H3T7 marker fragment after SspI digest showing presence of the *L*_*4*_ allele in homozygous state. R1—PI15923 possessing *L*_*3*_ allele, R2—PI260429 possessing *L*_*4*_ allele, S—susceptible genotype possessing L+ allele, 1–8 individuals of CAPS-80, M—DNA Ladder (Thermo Scientific Inc). Original, uncropped raw images are provided as supporting information in file S1 Raw images.

Analysis with PMFR11 and 087H3T7 markers revealed the presence of the *L*_*4*_ allele in CAPS-80. PCR with the PMFR11 marker amplified a fragment of 283 bp in all tested individuals of CAPS-80 corresponding to the susceptible allele in contrast to the control genotype (PI 159236) with an observed 269 bp fragment typical for the *L*_*3*_ allele (Fig 4A). PCR with 087H3T7 primers produced the expected 440 bp fragment (Fig 4B). No fragments were produced after cleavage of this amplicon with SspI in all individuals, indicative of the presence of *L*_*4*_ allele in the homozygotic state (*L*_*4*_*/L*_*4*_).

### Hierarchical cluster analysis

**Fig 5:**
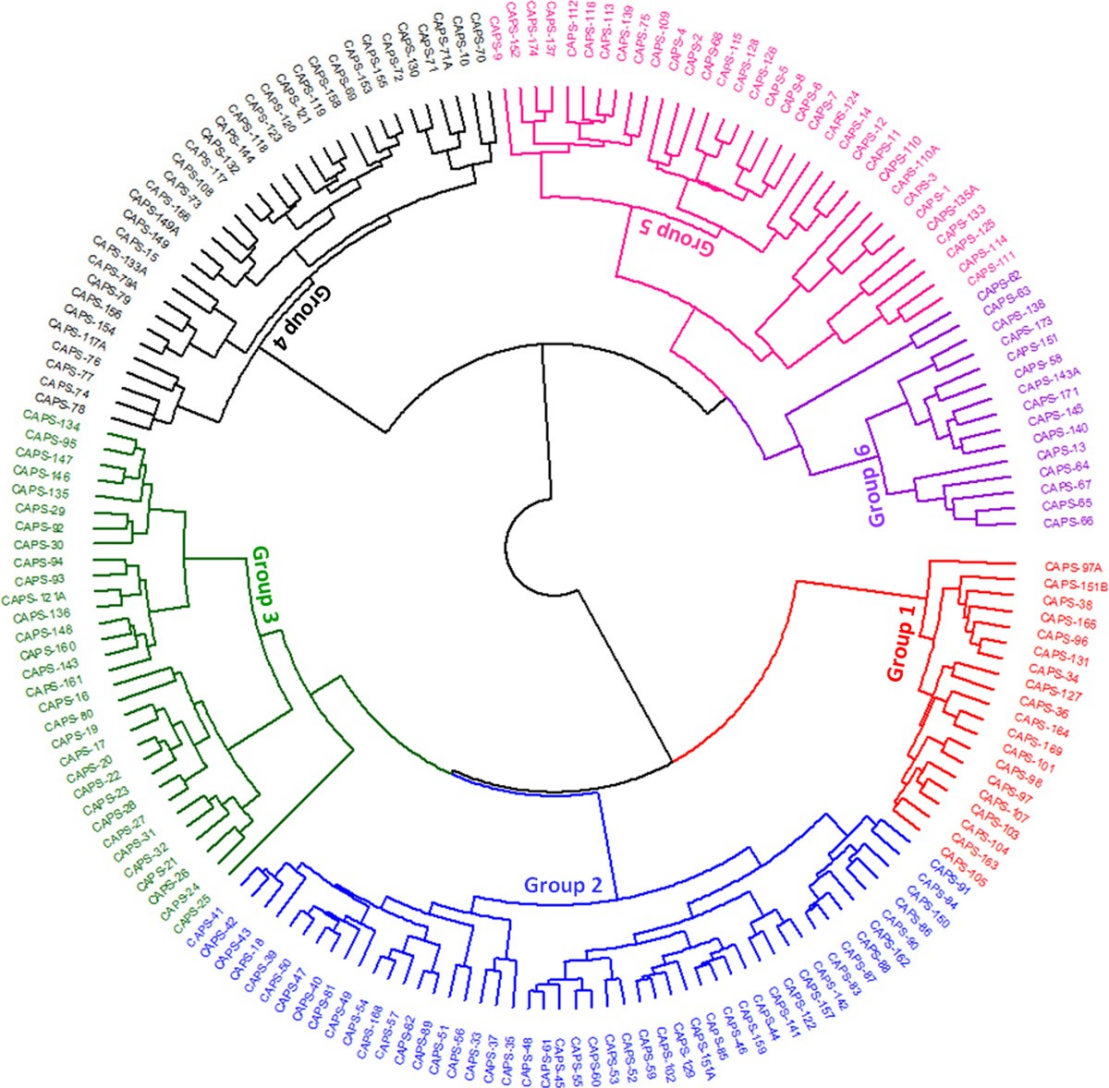
Hierarchical cluster analysis of evaluated pepper collection. Group 1, 2, 3, 4, 5, and 6 are assigned with red, blue, dark green, black, deep pink, and dark violet colors, respectively.

**Fig 6:**
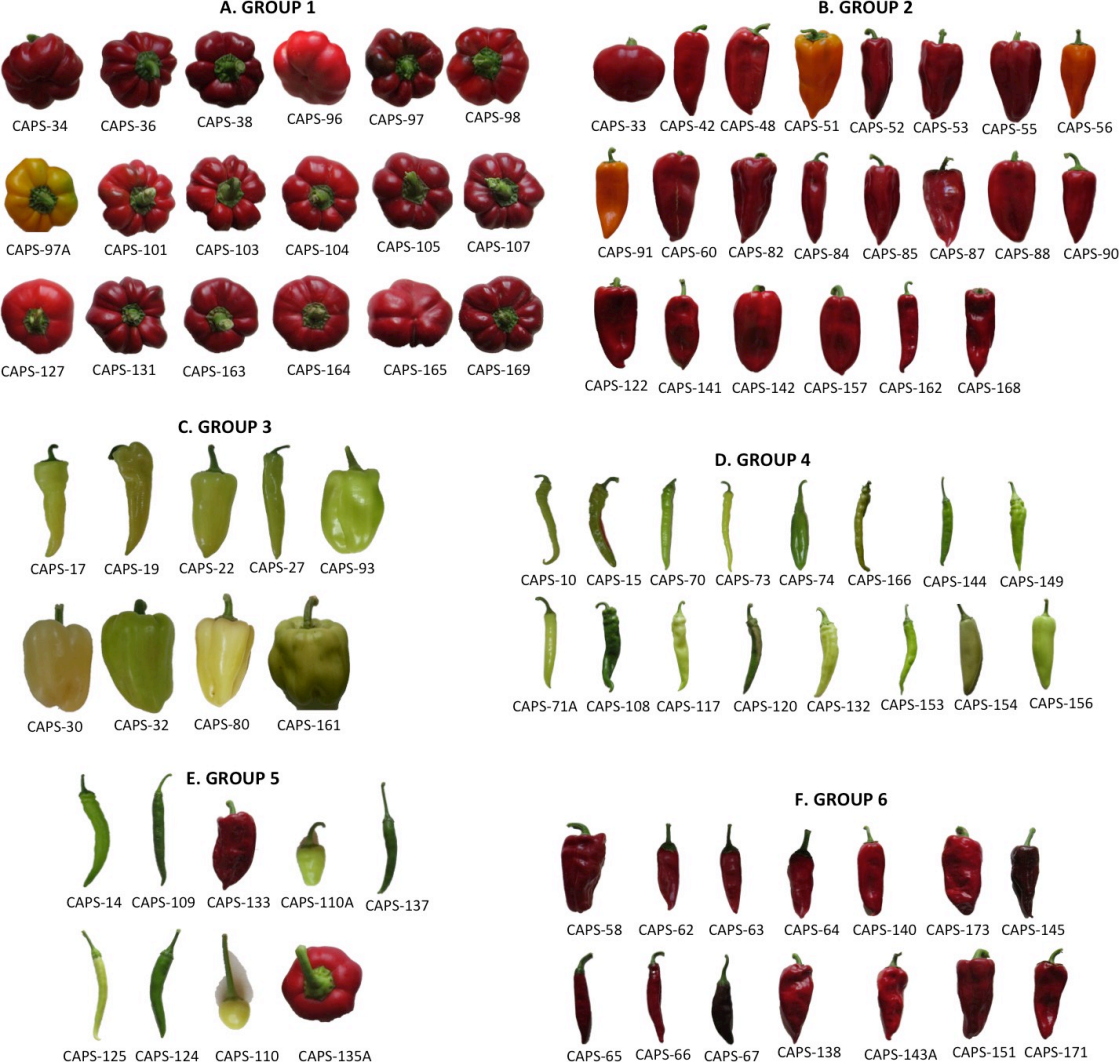
Phenotypic representation of fruits belonging to specific group/cluster alluded to the fruit morpho- and colorimetric characters.

Hierarchical clustering was done based on 32 agro-morphological and fruit quality traits. The Ward’s coefficient function was used to find the dissimilarity. A total of 180 accessions were classified into 6 distinct clusters based on their shape, size, color, and the way of usage (Fig 5 and S7 Table). Most accessions were populated into cluster 2 followed by clusters 4, 5, and 3. Only accessions belonging to specific cluster and also within the top 10% for selected traits of breeding interest are presented in Fig 6. Cluster 2 was mainly populated by Kapia accessions of big size fruits, conical shape with mostly red and few orange fruits (Figs 5 and 6B and S7 Table). Exceptions were of Pumpkin shape CAPS-33, 37 with red color large fruits and CAPS-18 of Sweet green type (Fig 6B). Accessions in cluster 4 were mainly from the Pungent group with medium fruit size, elongated shape, and green color (Fig 6D). However, 25% of populated accessions were represented by Sweet green type with medium to big size fruits, green before maturity and red and orange colors at maturity. Cluster 5 was also variable, but was mostly represented by accessions from the Pungent group, with small and medium fruits elongated to round shape, colored green before maturity and red at maturity stage. The exceptions were Sweet green accessions of medium (CAPS-11, 12, 14) to big (CAPS-133) fruit size and conical (CAPS-11, 12), elongated (CAPS-14) and blocky (CAPS-133) shape and red colored fruits at maturity stage (Fig 5 and S7 Table). Cluster 3 was populated by Sweet green accessions (Fig 6C) having mostly big size fruits of conical and bell or blocky shape, and green colored (Fig 6 and S7 Table). Accessions from clusters 1 and 6 were populated with Pumpkin shape (Fig 6A) and a mixture of Paprika and Kapia (Fig 6F), respectively. The fruits of Pumpkin shape were thicker and wider, flattened shape, colored red except CAPS-151A, which was of orange color (Fig 5 and S7 Table). Fruits from Paprika were thinner and narrow with an elongate and conical shape and red colored (Fig 5 and S7 Table) except CAPS-67, which had chocolate brown fruits. Clustering of distinct classes was characterized by accessions those were closely related and were populated within same clusters. This indicates that there was a clear separation of accessions based on their similarity in shape, size, and color except for cluster 4 (Fig 6D), with mixed accessions, representing Pungent and Sweet green, and cluster 6 (Fig 6F) populated predominantly with Paprika, and Kapia groups.

### Multivariate analysis

#### Principal component analysis

**Fig 7:**
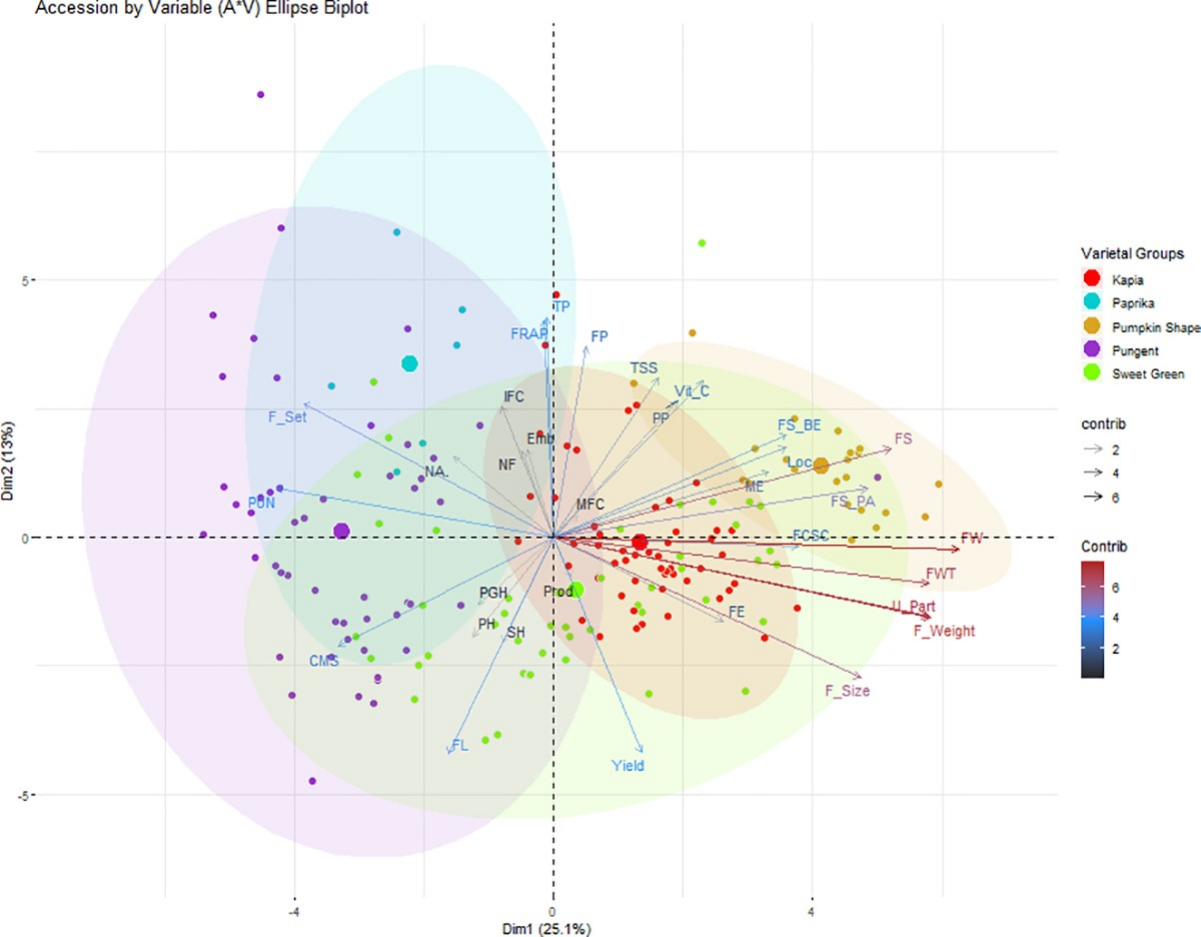
Accession by Variable (A*V) ellipse biplot. Accessions from Kapia, Paprika, Pumpkin Shape, Pungent, and Sweet Green are shown in red1, dark turquoise, goldenrod, darkorchid, and chartreuse color ellipses, respectively. Traits contributing to PC1 and PC2 are also assigned different gradient colors with a gradient ranging from 2,4, and 6 with gray15, dodgerblue, and firebrick, respectively. The traits included in PCA are abbreviated as PGH: Plant Growth Habit; PH: Plant Height; SH: Stem Height; NA: Nodal Anthocyanin; NF: Number of Flowers; Emb: Embranchment; CMS: Calyx Margin Shape; PP: Pedicel Position; FE: Flowering Earliness; ME: Maturity Earliness; F_Set: Fruit Set; FP: Fruit Position; FS: Fruit Shape; FS_PA: Fruit Shape at Peduncle Attachment; FS_BE: Fruit Shape at Blossom End; FSCS: Fruit Cross-Sectional Corrugation; F_Size: Fruit Size; IFC: Immature Fruit Color; MFC: Mature Fruit Color; PUN: Pungency; FL: Fruit Length; FW: Fruit Width; FWT: Fruit Wall Thickness; Loc: Locules; F_Weight: Fruit Weight; U_Part: Usable Part; Prod: Productivity; Vit C: Vitamin C; TP: Total Phenols; FRAP: Antioxidant Potential.

Eight major principal components (PCs) with >1 eigenvalue were the most meaningful components and contributed to the majority of cumulative variance (S8 Table). A total of 71% cumulative variance with respective contribution of PC 1–8 explained 25.11%, 13.05%, 11.17%, 5.98%, 5.05%, 4.05%, 3.41%, and 3.22% variance, respectively. The accession vs. variable biplot (A*V biplot) of PC1 and PC2 explained 38.16% variation (Fig 7) according to their fruit set, fruit position, shape, length, width, wall thickness, weight, usable part, yield, pungency, and fruit composition of TSS, Vit C, total phenols, and FRAP content (S8 Table). Inter-accession variation explained by PC1 and PC2 was 25.11% and 13.05% of the total variation, respectively (Fig 7).

The A*V ellipse biplot explained that if accessions were distinctly separated. If not what were those traits that overlapped the accessions from one varietal group with an ellipse of other varietal groups. Each ellipse was comprised of accessions belonging to a different varietal group and was assigned a specific color. Also traits contributing to PC1 and PC2 were also assigned different gradient colors. Characters associated with plant architecture and growth habit contributed the least variation (gradient <2) whereas fruit form traits contributed moderate variation (gradient >2 and > = 4). Moderate to highest variability of PC1 and PC2 was explained by fruit size, shape, and fruit composition of TSS, Vit C, and FRAP content (gradient of >4 and <6). Higher variation (gradient >6) was contributed by fruit width, wall thickness, weight, and usable part.

All pepper accessions were distributed across all quadrants and showed no distinct separation (Fig 7); however, the distribution of accessions belonging to specific varietal groups was populated in the specific quadrants. Accessions from Pumpkin shape (goldenrod ellipse) were populated in positive quadrant of PC1 and PC2 (Fig 7) and traits contributing variability to this quadrant were associated with early maturity, fruit shape, high Vit C, and TSS. Accessions from Paprika group (dark turquoise ellipse) were populated in quadrant 2 which comprised of the positive quadrant of PC2 and negative quadrant of PC1 where accessions spread in this quadrant seem to have high total phenols and FRAP. Most Pungent accessions (darkorchid ellipse) were spread across quadrant 2, and 3 where variation largely related to fruit pungency, immature fruit color, fruit length, and setting (Fig 7). Accessions belonging to Kapia (red ellipse) and Sweet green (chartreuse ellipse) were distributed across all four quadrants; however Sweet green varietal group seem to have higher variability than any other varietal group. Trait wise, fruit width, fruit wall thickness, fruit weight, usable part, and fruit shape mainly contributed positively to PC1 variability whereas fruit set and pungency contributed negatively (S9 Table and Fig 7). Fruit position and fruit compositional quality traits accounted positively for PC2 while fruit length and yield contributed negatively.

#### Correlation between different agro-morphological and fruit quality characters

The correlation matrix revealed positive and significant correlations between different yield components (Table 5 and S3 Fig); however, the correlations varied from weak to moderate. A significant positive correlation between fruit yield with fruit length (r = 0.296), width (r = 0.206), fruit wall thickness (r = 0.354), fruit weight (r = 0.330), usable part (r = 0.337) and productivity (r = 0.715) reflected that these traits were closely related; however, the correlations were low except for productivity per plant (Table 5 and S3 Fig). Apart from yield components, descriptors related to plant growth habit showed significant positive correlations with plant and stem height whereas negative correlation with maturity earliness, suggesting that erect plant growth habit usually results in late maturing tall plants. The correlations between flowering earliness and yield components were observed significantly positive, as would be expected (Table 5 and S3 Fig).

**Table 5:** Correlation coefficients among 22 agro-morphological characters among Balkan pepper collection.

	NA.	NF	PP	CMS	FP	IFC	MFC	FS	FS_PA	FS_BE	PUN	F_Size	FCSC	F_Set	FE	ME	Yld	PH	SH	Emb	FL	FW	FWT	Loc	Fwgt	UPart	Prod	TSS	Vit_C	TP	FRAP
**PGH**	0.08	0.08	-0.26***	0.21**	-0.15*	-0.14	-0.05	-0.19**	-0.16*	-0.10	0.31***	-0.08	0.05	0.11	0.07	-0.24***	-0.06	0.30***	0.39***	-0.11	0.13	-0.14	-0.18**	-0.20**	-0.14	-0.14	-0.05	0.02	-0.05	0.07	0.04
**NA.**		-0.03	-0.07	0.06	0.04	-0.06	0.01	-0.16*	-0.12	0.09	0.24***	-0.17*	-0.12	0.17*	-0.16*	-0.17*	-0.22**	0.19**	0.11	0.03	-0.18*	-0.20**	-0.25***	-0.05	-0.29***	-0.29***	-0.22**	0.04	0.01	0.12	0.06
**NF**			0.09	-0.06	0.21**	-0.05	0.02	-0.01	-0.13	-0.08	-0.07	-0.16*	-0.10	-0.05	0.01	-0.10	-0.21**	-0.18*	0.13	-0.18**	-0.06	-0.09	-0.17*	0.07	-0.13	-0.13	-0.18*	0.16*	0.17*	0.16*	0.15*
**PP**				-0.38***	0.35***	0.14	0.05	0.31***	0.36***	0.28***	-0.17*	0.04	0.05	-0.02	-0.11	0.18*	-0.11	-0.07	0.26***	0.19**	-0.31***	0.26***	0.22**	0.35***	0.16*	0.16*	-0.12	0.07	0.15*	0.08	0.02
**CMS**					-0.15*	-0.03	0.08	-0.45***	-0.49***	-0.31***	0.34***	-0.31***	-0.19**	0.20**	-0.09	-0.37***	0.01	0.23**	0.26***	-0.07	0.36***	-0.47***	-0.43***	-0.32***	-0.38***	-0.37***	-0.03	-0.19**	-0.26***	-0.07	-0.02
**FP**						0.17	0.02	0.33***	0.23**	0.45***	0.03	-0.19	0.03	0.15*	0.09	0.03	-0.20**	-0.16*	-0.25**	0.06	-0.49***	0.16**	0.08	0.32***	-0.01	-0.01	-0.20**	0.01	0.10	0.09	0.13
**IFC**							0.01	0.11	0.06	0.12	0.16*	-0.19**	-0.18*	0.25***	-0.08	-0.27***	0.13	-0.07	-0.25***	0.17*	-0.15*	-0.08	-0.02	0.14	-0.15*	-0.14	0.12	-0.41***	-0.25***	-0.24***	-0.15*
**MFC**								0.07	0.08	0.06	-0.03	0.06	0.02	-0.01	0.03	0.14	-0.13	0.13	0.22*	-0.02	-0.10	0.04	-0.01	0.07	0.01	0.01	-0.19*	0.01	-0.02	0.05	0.09
**FS**									0.68***	0.66***	-0.45 ***	0.52***	0.49***	-0.42 ***	0.23***	0.31***	0.06	-0.19**	0.21**	0.05	-0.48***	0.78***	0.69***	0.58***	0.60***	0.60***	0.05	0.12	0.28***	-0.01	0.02
**FS_PA**										0.52***	-0.45***	0.58***	0.41***	-0.39***	0.26***	0.25***	0.11	-0.16*	-0.13	0.08	-0.33***	0.77***	0.64***	0.49***	0.64***	0.64***	0.18*	0.09	0.21**	-0.04	-0.10
**FS_BE**											-0.14	0.17*	0.37***	-0.09	0.14	0.09	-0.08	0.05	0.22**	0.08	-0.67***	0.51***	0.43***	0.63***	0.25***	0.25***	-0.15*	-0.12	0.02	-0.10	-0.09
**PUN**												-0.59***	-0.33***	0.63***	-0.27***	-0.42***	-0.20**	0.28***	0.04	0.05	-0.06	-0.60***	-0.55***	-0.31***	-0.61***	-0.61***	-0.23**	-0.30***	-0.33***	-0.04	0.06
**F_Size**													0.41***	-0.71***	0.38***	0.34***	0.24***	-0.15*	0.10	-0.09	0.16*	0.76***	0.72***	0.19**	0.81***	0.81***	0.33***	0.25***	0.23**	-0.08	-0.10
**FCSC**														0.42**	0.22**	0.22**	0.07	0.01	0.03	-0.01	0.02	0.53***	0.38***	0.25***	0.50***	0.50***	0.07	0.20**	0.24**	0.11	0.09
**F_Set**															-0.48**	-0.22**	-0.15*	0.13	-0.12	0.13	-0.28***	-0.61**	-0.55***	-0.19**	-0.67***	-0.67***	-0.21**	-0.29***	-0.23**	0.04	0.07
**FE**																0.03	0.21**	-0.02	0.10	-0.07	0.10	0.38***	0.34***	0.21**	0.41***	0.41***	0.20**	0.07	-0.03	-0.10	-0.15*
**ME**																	-0.04	-0.19**	-0.10	-0.11	-0.15*	0.41***	0.43***	0.18*	0.46***	0.45***	-0.01	0.52***	0.49***	0.34***	0.28***
**Yield**																		0.01	-0.11	-0.10	0.30***	0.21**	0.35***	0.05	0.33***	0.34***	0.72***	-0.30***	-0.27***	-0.45***	-0.44***
**PH**																			0.46***	0.10	0.05	-0.17*	-0.21**	-0.11	-0.15*	-0.17*	-0.12	-0.18**	-0.24***	-0.15*	-0.16*
**SH**																				-0.02	0.33***	-0.09	-0.21**	-0.3***	-0.04	-0.05	-0.14	0.14	-0.01	0.09	0.04
**Emb**																					-0.16*	0.03	-0.04	0.10	-0.05	-0.04	-0.09	-0.05	-0.03	0.10	0.06
**FL**																						-0.24**	-0.21**	-0.55**	0.06	0.07	0.38***	-0.01	-0.19**	-0.14	-0.13
**FW**																							0.90***	0.54***	0.90***	0.90***	0.26***	0.24***	0.30***	-0.04	-0.09
**FWT**																								0.48***	0.90***	0.89***	0.38***	0.16*	0.25***	-0.13	-0.15*
**Loc**																									0.34***	0.34***	0.01	0.03	0.13	-0.05	-0.12
**Fwgt**																										0.99***	0.41***	0.29***	0.30***	-0.07	-0.13
**UPart**																											0.41***	0.28***	0.29***	-0.08	-0.14
**Prod**																												-0.22**	-0.23***	-0.41***	-0.43***
**TSS**																													0.77***	0.78***	0.65***
**Vit_C**																														0.67***	0.61***
**TP**																															0.88***

*, **, *** correlation coefficient at significant level of P<0.05, respectively. PGH: Plant Growth Habit; NA: Nodal Anthocyanin; NF: Number of Flowers; PP: Pedicel Position; CMS: Calyx Margin Shape; FP: Fruit Position; IFC: Immature Fruit Color; MFC: Mature Fruit Color; FS: Fruit Shape; FS_PA: Fruit Shape at Peduncle Attachment; FS_BE: Fruit Shape at Blossom End; Pun: Pungency; F_Size: Fruit Size; FE: Flowering Earliness; ME: Maturity Earliness; PH: Plant Height; SH: Stem Height; Emb: Embranchment; FL: Fruit Length; FW: Fruit Width; FWT: Fruit Wall Thickness; Loc: Locules; Fwgt: Fruit Weight; UPart: usable Part; Prod: Productivity; Vit_C: Vitamin C; TP: Total Phenols; FRAP: Ferric Reducing Antioxidant Power

The fruit quality concerning TSS, Vit C, total phenols, and FRAP content showed significant positive correlations where TSS strongly correlated with Vit C (r = 0.772), total phenols (r = 0.777), and FRAP (r = 0.647) whereas Vit C correlated with total phenols (r = 0.669) and FRAP (r = 0.607) while total phenols and FRAP was highly correlated (r = 0.877) among all fruit quality traits (Table 5 and S3 Fig). Specific to each quality trait, TSS content displayed a positive correlation with maturity earliness, fruit size, width, and weight whereas negatively correlated with immature fruit color, pungency, fruit set, yield, and productivity; however, correlations were measured poor. The Vit C, total phenols, and FRAP discerned similar trends, where significant positive correlations with maturity earliness and negative correlations with immature fruit color, flowering earliness, fruit wall thickness, yield, and productivity (Table 5 and S3 Fig). However, all correlations between agronomic and fruit quality traits were weak except for a moderate correlation of maturity earliness with TSS (r = 0.517), and Vit C (r = 0.487).

#### Correlation network

**Fig 8:**
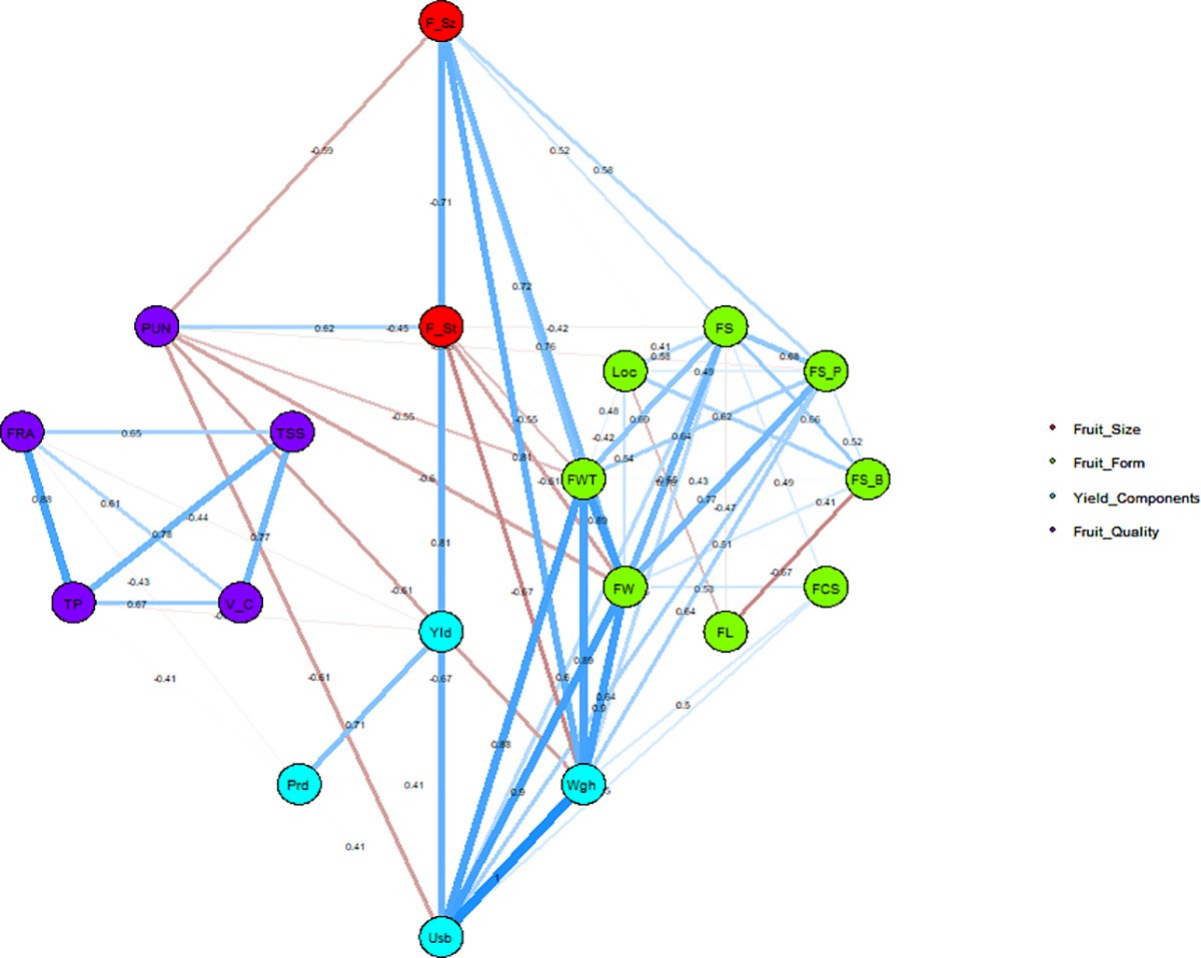
Correlation network of selected 19 plant architectural, fruit form, yield components, and fruit quality traits considered to construct the correlation network (r = 0.4). Traits contributing to PC1 and PC2 are also assigned different gradient colors with gradient ranging from 2, 4, and 6 with gray15, dodgerblue, and firebrick, respectively. Width of correlation band explains the correlation robustness while colors red and dodgerblue illustrates negative and positive correlation, respectively. The traits included in PCA are abbreviated as F_St: Fruit Set; F_Sz: Fruit Size; PUN: Pungency; FS: Fruit Shape; FS_P: Fruit Shape at Pedicel Attachment; FS_B: Fruit Shape at Blossom End; FCS: Fruit Cross-Sectional Corrugation; FL: Fruit Length; FW: Fruit Width; FWT: Fruit Wall Thickness; Loc: Locules; Yld: Fruit Yield; Wgh: Fruit Weight; Usb: Usable Part; Prd: Productivity; Brx: Brix; V_C: Vitamin C; TP: Total Phenols; FRP: Antioxidant Potential.

The association between closely related traits was investigated using a correlation network (Fig 8). Only traits with a correlation >0.4 were considered during correlation network construction. A total of 19 descriptors belonging to fruit form, yield components, and fruit quality were used to find the interrelatedness between closely related traits. Correlation robustness was explained by the width of the correlation band, and the colors red and dodgerblue illustrated negative and positive correlation, respectively. Most closely interrelated traits were positively correlated except for fruit size, fruit setting, and pungency. Although fruit size was negatively correlated with fruit set and yield components, it also showed positive correlation with fruit shape (Fig 8), while most negative inter relatedness was seen between pungency with fruit form and yield components. Fruit quality traits of TSS, Vit C, total phenols, and FRAP discerned strong relatedness among themselves while neither showed any relationship with fruit form and yield components except for a negative relationship of FRAP with yield and productivity (Fig 8). As anticipated, most traits belonging to fruit form and fruit quality showed strong relationships within group and between categories except for fruit corrugation.

### Directional selection of superior accessions for the fruit agro-morphological, productivity, and fruit quality traits

In order to identify the best performing candidate accessions, yield components (fruit yield per plant, fruit size, fruit weight, fruit length, fruit width, and productivity per plant), and fruit compositional traits were major traits of interests and were taken into the account for further selection. Among the 180 tested pepper accessions, 18 accessions were selected based on yield components (Table 6A) and fruit compositional traits (Table 6B). Some accessions discerning higher values for agronomic traits were also reflected in higher fruit quality such as CAPS-14 commonly present in fruit length, productivity, and FRAP (highlighted yellow in Table 6A and 6B). While accessions CAPS-34, 103 were also commonly seen in fruit width, wall thickness, fruit weight, and Vit C (highlighted gray in Table 6A and 6B). Accessions CAPS-10, 14, 17, 70, and 154 with longer fruit length also had higher productivity per plant (highlighted yellow in Table 6A). Interestingly, accessions CAPS-34, 96, 97, 98, 101, 103–105, 122, 131, 142, 163–165, and CAPS-169 with broader fruit width had thicker fruit wall and higher fruit weight (highlighted gray in Table 6A). Only CAPS-80 was seen to have thicker fruit wall with higher fruit weight, and productivity (highlighted pink in Table 6A). Variability found for fruit length, width, wall thickness, fruit weight, and productivity was significantly lower among the top 10% selected accessions (Table 6A) in comparison to variability reported among total collection (Table 1A).

**Table 6:** Performance statistics of top 10% accessions for fruit form, morphology (6A), and fruit quality (6B) traits.

	A. Fruit Form and Morphology									B. Fruit Compositional Quality							
Accession	FL	Accession	FW	Accession	FWT	Accession	F_Wt	Accession	Prod	Accession	TSS	Accession	Vit C	Accession	TP	Accession	FRAP
CAPS-73	22.75	CAPS‐98	8.65	CAPS‐105	6.66	CAPS-157	180.64	CAPS‐70	1.26	CAPS-138	18.07	CAPS-33	230.93	CAPS‐138	414.78	CAPS‐138	26.85
CAPS-166	22.67	CAPS‐103	8.58	CAPS‐103	5.96	CAPS‐142	178.48	CAPS-42	1.05	CAPS‐173	16.10	CAPS‐34	218.67	CAPS-64	295.84	CAPS‐133	21.76
CAPS-108	19.04	CAPS‐104	8.08	CAPS-33	5.84	CAPS‐103	151.82	CAPS‐10	0.96	CAPS‐133	15.27	CAPS-36	236.27	CAPS‐133	277.34	CAPS-135A	20.21
CAPS‐14	18.44	CAPS‐34	7.98	CAPS‐131	5.83	CAPS‐104	148.11	CAPS-156	0.95	CAPS-145	13.83	CAPS-51	227.73	CAPS‐173	272.36	CAPS‐173	18.84
CAPS‐154	18.17	CAPS‐164	7.88	CAPS-127	5.75	CAPS‐98	145.48	CAPS-74	0.93	CAPS-64	13.30	CAPS-56	231.47	CAPS‐151	237.67	CAPS‐140	17.08
CAPS-132	18.11	CAPS‐169	7.69	CAPS‐98	5.72	CAPS-60	139.8	CAPS-88	0.88	CAPS-143A	12.53	CAPS-58	260.80	CAPS‐140	230.24	CAPS‐143A	16.63
CAPS‐70	17.69	CAPS‐163	7.68	CAPS‐104	5.71	CAPS-141	138.6	CAPS-53	0.86	CAPS-171	12.50	CAPS‐62	249.60	CAPS‐143A	229.13	CAPS‐164	16.08
CAPS-15	17.65	CAPS‐101	7.61	CAPS‐163	5.65	CAPS‐97	137.41	CAPS-52	0.86	CAPS-140	12.47	CAPS‐63	225.07	CAPS‐62	227.81	CAPS-124	15.86
CAPS-153	17.63	CAPS‐105	7.59	CAPS‐96	5.58	CAPS‐34	137.38	CAPS-84	0.83	CAPS-66	12.43	CAPS-90	193.07	CAPS-65	220.63	CAPS‐171	15.46
CAPS‐10	17.24	CAPS-107	7.55	CAPS‐80	5.54	CAPS-48	133.88	CAPS‐80	0.78	CAPS-65	12.37	CAPS-91	213.87	CAPS‐171	218.75	CAPS‐62	14.99
CAPS‐17	17.07	CAPS‐131	7.54	CAPS-36	5.5	CAPS‐122	131.69	CAPS-22	0.77	CAPS‐62	11.57	CAPS‐103	192.03	CAPS-67	212.61	CAPS-127A	13.97
CAPS-117	16.83	CAPS‐97	7.38	CAPS‐164	5.49	CAPS‐105	129.59	CAPS-87	0.76	CAPS‐151	11.53	CAPS‐133	224.33	CAPS‐110	210.40	CAPS-107	13.84
CAPS-149	16.78	CAPS‐142	7.37	CAPS-97A	5.43	CAPS-55	129.08	CAPS‐17	0.76	CAPS-58	11.33	CAPS‐151	229.27	CAPS‐63	203.35	CAPS-127	12.91
CAPS-120	16.62	CAPS-32	7.08	CAPS-30	5.34	CAPS‐131	128.85	CAPS‐14	0.76	CAPS-67	10.73	CAPS-161	197.23	CAPS-73	201.02	CAPS‐14	12.65
CAPS-162	16.27	CAPS‐165	7.05	CAPS‐34	5.32	CAPS‐80	127.99	CAPS-85	0.76	CAPS-91	10.50	CAPS-166	273.47	CAPS-66	192.49	CAPS-125	12.62
CAPS-19	16.03	CAPS‐96	6.94	CAPS-38	5.32	CAPS‐101	124.64	CAPS‐154	0.74	CAPS‐63	10.43	CAPS‐173	201.80	CAPS-145	185.22	CAPS‐110	12.38
CAPS-71A	15.92	CAPS‐122	6.88	CAPS‐169	5.31	CAPS-82	123.93	CAPS-27	0.74	CAPS-56	10.07	CAPS-127A	202.57	CAPS-137	182.50	CAPS-110A	12.15
CAPS-144	15.83	CAPS-93	6.86	CAPS‐165	5.17	CAPS‐164	123.58	CAPS‐163	0.74	CAPS-162	9.77	CAPS-135A	194.20	CAPS-168	181.58	CAPS-109	12.09
**Group Mean**	17.82		7.58		5.62		139.50		9.81	**Group Mean**	12.49		222.35		232.98		15.91
**Range**	15.83–22.75		6.86–8.65		5.17–6.66		123.58–180.6		0.74–1.26	**Range**	9.77–18.07		192.03–230.93		181.8–414.8		12.09–26.85
**SD**	1.89		0.50		0.32		15.80		0.13	**SD**	2.08		22.21		52.76		3.72
**SE**	0.444		0.117		0.076		3.725		0.030	**SE**	0.490		5.235		12.44		0.878
**Group CV (%)**	10.58		6.57		5.71		11.33		15.12	**Group CV (%)**	16.63		9.99		22.64		23.41
**Pop Mean**	10.25		4.35		3.45		66.62		0.55	**Pop Mean**	7.24		115.59		125.28		7.27
**Pop CV (%)**	21.54		13.41		19.94		24.40		33.90	**Pop CV (%)**	46.11		61.12		51.06		84.23

CV: Coefficient of Variation; SD: Standard Deviation; SE: Standard Error; FL: Fruit Length; FW: Fruit Width; FWT: Fruit Wall Thickness; Vit C: Vitamin C; TP: Total Phenols; FRAP: Ferric Reducing Antioxidant Power

Selected accessions with higher fruit composition for one trait were often also seen to have higher composition for other traits such as accessions CAPS-173, 133, and 62, which were present across all compositional traits of TSS content, Vit C, total phenols, and FRAP (highlighted red in Table 6B) while CAPS-151 and 63 were present in TSS, Vit C, and total phenols (highlighted light green in Table 6B) whereas CAPS-138, 140, 143A, 171, and 110 present in total phenols and FRAP content (highlighted turquoise in Table 6B), respectively. The variability found for Vit C (115.59%) and total phenols (125.28%) was higher among selected top 10% accessions (Table 6B) in comparison to variability reported for Vit C (61.12%) and total phenols (51.06%) among total collection (Table 2A). Variability seen among top 10% selected accessions (Table 6B) was lower for TSS (16.63%) and FRAP (23.41%) content in comparison to total collection’s TSS (46.11%) and FRAP (84.23%) content (Table 2A).

## Discussion

### Agronomical, fruit quality, and virus screening traits characterization and variation within and between accessions

All agro-morphological, yield components, and fruit compositional traits displayed significant genotypic differences, which suggest that the Balkan pepper collection comprises considerable phenotypic diversity and genotypic variability which might be useful for pre-breeding and variety development efforts (Tables 1 and 2). The A*Y interaction clearly presented the traits that have stable expression across years and identified the accessions that are stable and performing consistently across years. Genotypic variation and biomorphological diversity observed in this study is in alignment with previous work on *C*. *annuum* intra-species phenotypic variability [9,11,27,33,49–50] and biochemical variability of total phenols between varieties of *C*. *annuum* and *C*. *frutescence* species [51]. The ANOVA for agro-morphological traits displayed highly significant differences within and between years revealing their higher level of variability. Our findings are in agreement with other studies [31,52]; however, the year effects were not significant in Occhiuto et al [31].

The diversity observed through a broad range of variability measured for 24 agro-morphological (S1 Fig), four yield components (Fig 3), and four fruit quality traits (Fig 4) revealed appreciable biomorphological variation in the studied collection, which is in concurrence with previous studies using agronomic [45,50], fruit morphology [53], and fruit quality [51,54] traits to characterize the pepper germplasm. Variability found in traits associated with fruit shape, compositional quality, and yield components might be useful for broader acceptability of pepper collection for cultivation [49–50], consumer preference [55–56], use in local cuisine [51], processing [57], packaging [58], and export. Variability observed for fruit size, shape, and color indicate that the Balkan pepper producers and end-users in different regions might prefer fruits with particular forms, leading to varietal groups development in those regions as seen in Argentinian and Spanish pepper varietal typification [31,33]. Besides fruit form, the growth habit is also an important determinant in the adaptation and cultivation in different environment, particularly of compact or erect peppers [50]. There has not been a major study that measures pepper diversity across all pepper-producing Balkan regions, other than a handful of small studies investigating agronomical, genetic, and biochemical diversity [9,26,37,59].

Besides high variability identified for agro-morphological descriptors, a noticeable variability was observed for fruit quality between accessions. Within-group fruit quality was higher in Kapia, Pumpkin type, and Paprika than Pungent and Sweet green types. However, this was due to the fact that fruits analyzed from the latter two groups were harvested before maturity, thus accumulation of TSS, Vit C, TP, and FRAP might have been lower than those harvested at maturity [37].

Conventionally, virologists and breeders have counted on four different ‘L’ locus alleles (*L*_*1*_-*L*_*4*_) as a resistance source against tobamoviruses [44,60,61]. The implication of the co-dominant markers in our case allowed discrimination between the *L*_*3*_ and *L*_*4*_ alleles in CAPS-80 that have the same phenotype after challenging with only one pathotype not able to identify the allele with higher resistance [62]. Application of PMFR1 marker was found to be highly useful in identifying resistant allele against tobamoviruses as similarly reported by Hudcovicová et al [63] study of Hungarian cultivars. Overall, variability information obtained for myriad traits is important for future breeding efforts in developing lines and varieties that possess the desired fruit features, virus resistance, high yield, and enhanced bioactive compounds.

### Hierarchical cluster analysis

Groups obtained using agglomerative hierarchical clustering were consistent with pre-defined varietal groups (Fig 5), which suggest that the accessions with similar plant and fruit traits (Fig 6) were grouped together [9]. Clusters 4 and 6 were exceptions to this likely because the measured traits have close relatedness and might have subtle differences only [33,58]. Distinct clusters were not established by the inclusion of accessions collected from the similar growing environments that is indicative of unhindered seed exchange across the Balkan region, as observed previously in a Brazilian *Capsicum* collection [45,64]. Hierarchical clustering also elaborated on the role of plant architectural, fruit shape, size, form, color, and maturity traits in the assessment of plant and fruit diversity and varietal typification as previously reported by morphometric and colorimetric descriptors of TA [33,58,65]. Clear group separation using Ward’s coefficient indicates that within varietal group variability is well characterized and defined as previously explained in *C*. *annuum*, *baccatum*, and *chinense* intra-species variability [30].

### Principal component analysis

PCA was employed to identify the divergence among accessions as a measure of phenotypic and fruit compositional variability, and to investigate the relative contribution of different biomorphological characters to the total diversity [19,45]. The PCA results further supported the presence of biomorphological diversity and variability recorded in ANOVA. Eigenvector derived PCs suggested that plant and stem height, fruit shape, width, wall thickness, fruit weight, and fruit quality traits of TSS, Vit C, total phenols, and FRAP were most discriminative traits and could be effective in the characterization of Balkan pepper accessions (Fig 7 and S8 Table). Ballina-Gomez et al [27], Bozokalfa and Esiyok [55], Nsabiyera et al [49] and Tsonev et al [9] have made similar observations and underlined the differential contribution and influence of different morphological traits in contributing to total variability and its role in germplasm characterization. The role of biomorphological traits in comprehending divergence among pepper populations was clearly seen in our study and has previously been reported across trans-continental *Capsicum* studies with noted observations made in Brazilian *C*. *chinense* [45], Turkish pepper collection [26], Ugandan hot pepper [49], 19 Bulgarian accessions [9], and Serbian *Capsicum* collection [66]. Similarly, the usefulness of fruit morphometric and colorimetric descriptors has also been proven to be useful in characterizing fruit diversity in similar *Capsicum* collection as presented in this study [38] as well as in unrelated pepper collections from Italy [65], and Spain [33].

### Relationship among fruit form, yield components, and fruit quality

Here only the moderate to highly correlated traits (r = > 0.40) included in correlation network analysis were the subjects of discussion. No meaningful correlation was seen among growth habit and plant architectural traits except for fruit shape at blossom end (FS_BE) and fruit position (Table 6 and S2 Fig), which is in contrast with the morphological characterization of *C*. *annuum* collection of Northern Benin [67] and Spain [33]. Strongly significant and positive correlations between fruit form characters indicate that the one trait may influence the expression of the other traits such as seen between fruit size, width, wall thickness, fruit weight, and yield components. It could be assumed that there is likely a genetic linkage and non-additive gene interaction [31,52,68,69]. On the other hand, correlation of fruit length with fruit width and wall thickness was significantly negative and was in accordance with earlier observations of Tsonev et al [9]. However, these results are in contrast with Matthew et al [70] and Ullah et al [52], which reported positive correlations for the same. The strongly positive correlation reported between fruit weight and usable part is in accordance with the strong correlation reported in Todorova et al [71] red pepper study. A noticeable significant positive correlation was seen between traits associated with fruit form and Vit C; however, the correlation was weak and no such observation has previously been reported yet to our knowledge. A significant positive correlation among yield component and fruit quality of TSS and Vit C suggest that these accessions could be considered for future pre-breeding so that it can further expedite variety development and reduce the cost required for additional evaluation [19,49]. A correlation-based descriptors network further validated the relationship observed between trait categories as well as demonstrated the relatedness within fruit size, form, yield components, and fruit quality traits.

### Directional selection of best performing accessions

Selection of high yielding and better performing accessions under multi-year trial was also one of the aims of this study. The top 10% of the best performing candidates that displaying polymorphism for phenotypic variability (Table 6) were selected so that we can concentrate our future pre-breeding efforts to create improved breeding lines and establish the pepper breeding pipeline. Seven traits related to corolla and seed were monomorphic and can be removed in a future germplasm characterization as indicated by Carvalho et al [50] study of intraspecific characterization of *C*. *frutescence*.

Relatedness and dependency among traits showed that the selection of accession with higher fruit length and width would result in the selection of bigger fruits. This correlation has shown in previous *Capsicum* studies leading to greater fruit weight [49] and fruit yield [72]. This complementation of interrelated traits is mainly due to the fact that these traits are complementary to each other and contributes to yield components [28,72–73]. Accessions with higher fruit length and productivity should be considered when breeding for high yield. The accessions with broader fruit width, fruit wall thickness, and fruit weight can be utilized to breed peppers for stuffing or roasting purposes.

The accessions identified for desirable fruit shape, high yield, and enhanced TSS, Vit C, total phenols, and FRAP content might have a favorable socio-economic impact on producers and other stakeholders involved in pepper production and processing. Accessions performing better for one or more traits can be used for future pre-breeding purposes and be included in multi-location and year studies for further evaluation. Once promising lines are identified, they can be subsequently registered or patented as new breeding lines or varieties. The prospective breeding lines or varieties will be made available to the European breeding community under Bulgarian and European seed transfer agreements.

## Supporting information

S1 Raw images Original, uncropped raw images of stained gels supporting all gel results. (PDF)

S1 Fig Frequency distribution of plant architectural, morphological, fruit form and yield components of Balkan pepper collection. (XLSX)

S2 Fig Representative symptoms of TMV, PMMoV, and TSWV. **(A)** Necrotic local lesion, **(B)** net like necrosis, **(C)** leaf abscission, and **(D)** vein clearing and chlorosis after inoculation with TMV; **(E)** Necrotic local lesions 3 days after inoculation with PMMoV in detached leaf test; **(F)** mosaic and diffuse chlorotic spots 6–8 dpi with TSWV.
(TIF)

S3 Fig Tripartite traits correlation matrix heatmap. (TIF)

S1 Table Descriptive statistics and Analysis of Variance (ANOVA) of fruit agro-morphological and productivity traits within Varietal Groups (VGs) evaluated across years. (DOCX)

S2 Table Descriptive statistics and Analysis of Variance (ANOVA) of fruit agro-morphological and productivity traits within Varietal Groups (VGs) evaluated during 2018. (DOCX)

S3 Table Descriptive statistics and Analysis of Variance (ANOVA) of fruit agro-morphological and productivity traits within Varietal Groups (VGs) evaluated during 2019. (DOCX)

S4 Table Accessions pooled mean for agro-morphological traits. (XLSX)

S5 Table Accessions pooled mean for fruit quality traits. (XLSX)

S6 Table Balkan *Capsicum* collection response to TMV, PMMoV, and TSWV screening. (XLSX)

S7 Table Cluster analysis grouping. (XLSX)

S8 Table PCA eigenvalue, contribution of each PC %variance, and cumulative variance. (XLSX)

S9 Table Variable contribution and correlation coefficient (R^2^) of first five PCs. (XLS)
