# Supplementary material for: A step towards Balkan Capsicum annuum L. core collection: Phenotypic and biochemical characterization of 180 accessions for agronomic, fruit quality, and virus resistance traits
Source: PLoS One. 2020 Aug 17;15(8):e0237741. doi: 10.1371/journal.pone.0237741 (PMC7430755; doi:10.1371/journal.pone.0237741)
Supplement: S1 Raw images — (PDF) [file pone.0237741.s001.pdf]

S1_raw_images

A step towards Balkan *Capsicum annuum* L. core collection: Phenotypic and biochemical characterization of 180 accessions for agronomic, fruit quality, and virus resistance traits

Amol N. Nankar[1*], Velichka Todorova[2], Ivanka Tringovska[2#], Gancho Pasev[2#], Vesela Radeva-Ivanova[2‡], Valentina Ivanova[1‡], Dimitrina Kostova[1‡]

[1] Center of Plant Systems Biology and Biotechnology (CPSBB), 139 Ruski Blvd, Plovdiv 4000, Bulgaria

[2] Maritsa Vegetable Crops Research Institute (MVCRI), 32 Brezovsko Shosse Str, Plovdiv, 4003, Bulgaria

*** Corresponding author**

Email: nankaramol@gmail.com; nankar@cpsbb.eu (ANN)

The PCR products were separated on 2% agarose gel for 300 min at 80 V and stained with SimplySafe™ (EURx Ltd., Poland). The images were taken with Biovision+115, Vilber Lourmat and exported as high-resolution TIFF files using Vision Capt++ software v.12.9.

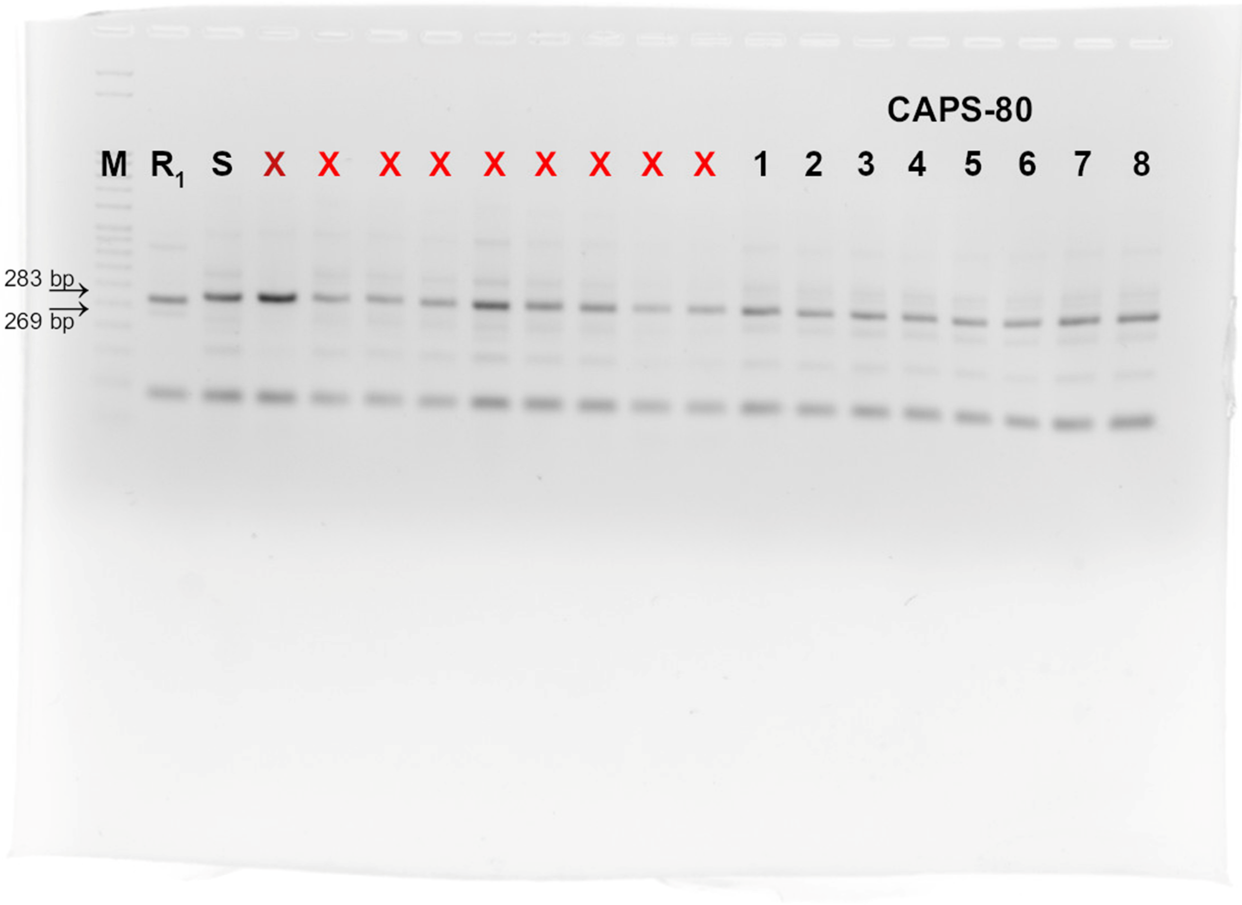

Uncropped image of the gel presented in Fig. 4A.

The image was cropped and the unrelated lanes marked with a red X were excluded. The splicing was denoted by vertical black line on the Fig. 4A The contrast of the whole image was slightly adjusted to improve the visibility of the bands.

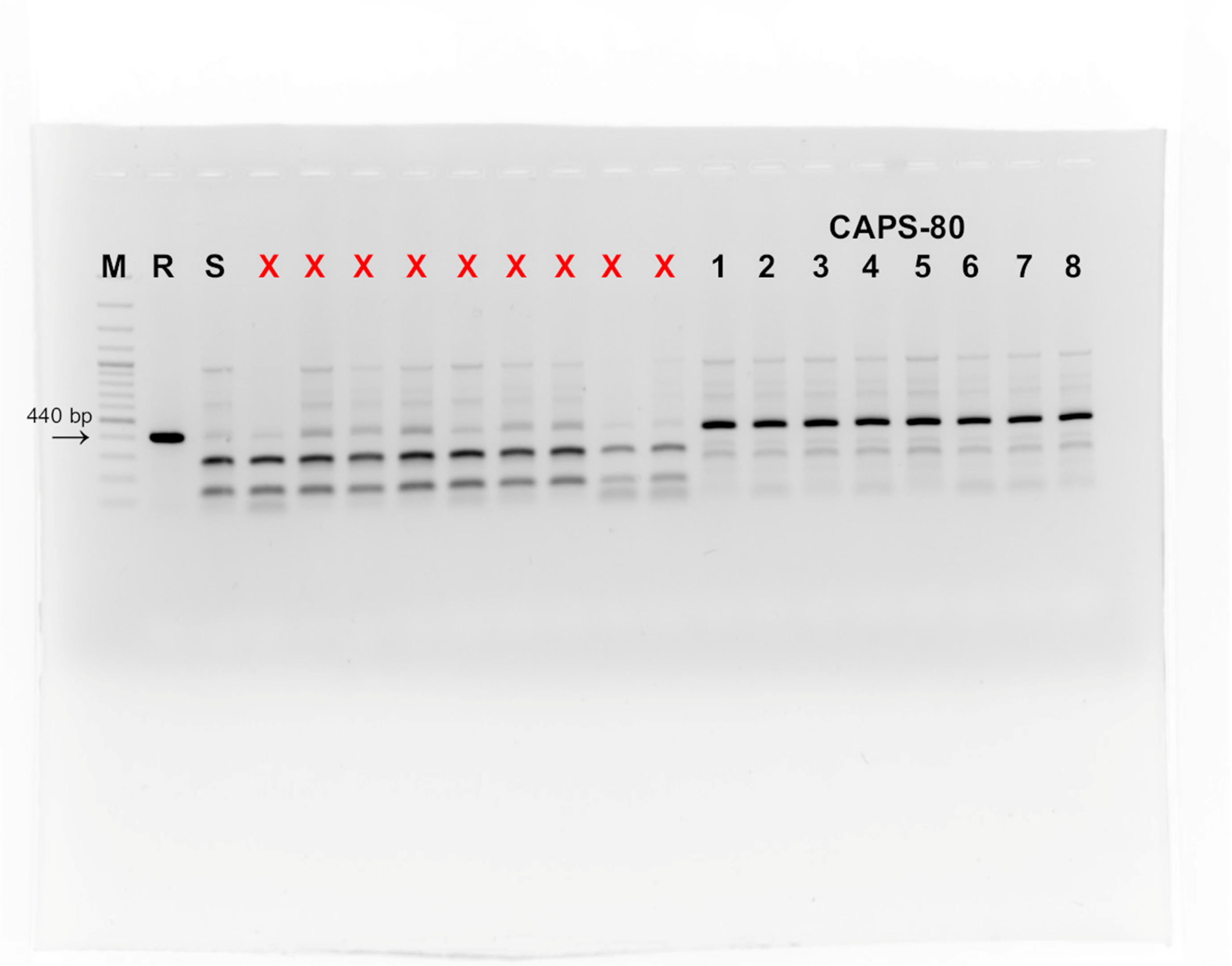

Uncropped image of the gel presented in Fig. 4B.

The image was cropped and the unrelated lanes marked with a red X were excluded. The splicing was denoted by vertical black line on the Fig. 4B. The contrast of the whole image was slightly adjusted to improve the visibility of the bands.
