# Supplementary figures and images for: A step towards Balkan Capsicum annuum L. core collection: Phenotypic and biochemical characterization of 180 accessions for agronomic, fruit quality, and virus resistance traits
Source: PLoS One. 2020 Aug 17;15(8):e0237741. doi: 10.1371/journal.pone.0237741 (PMC7430755; doi:10.1371/journal.pone.0237741)

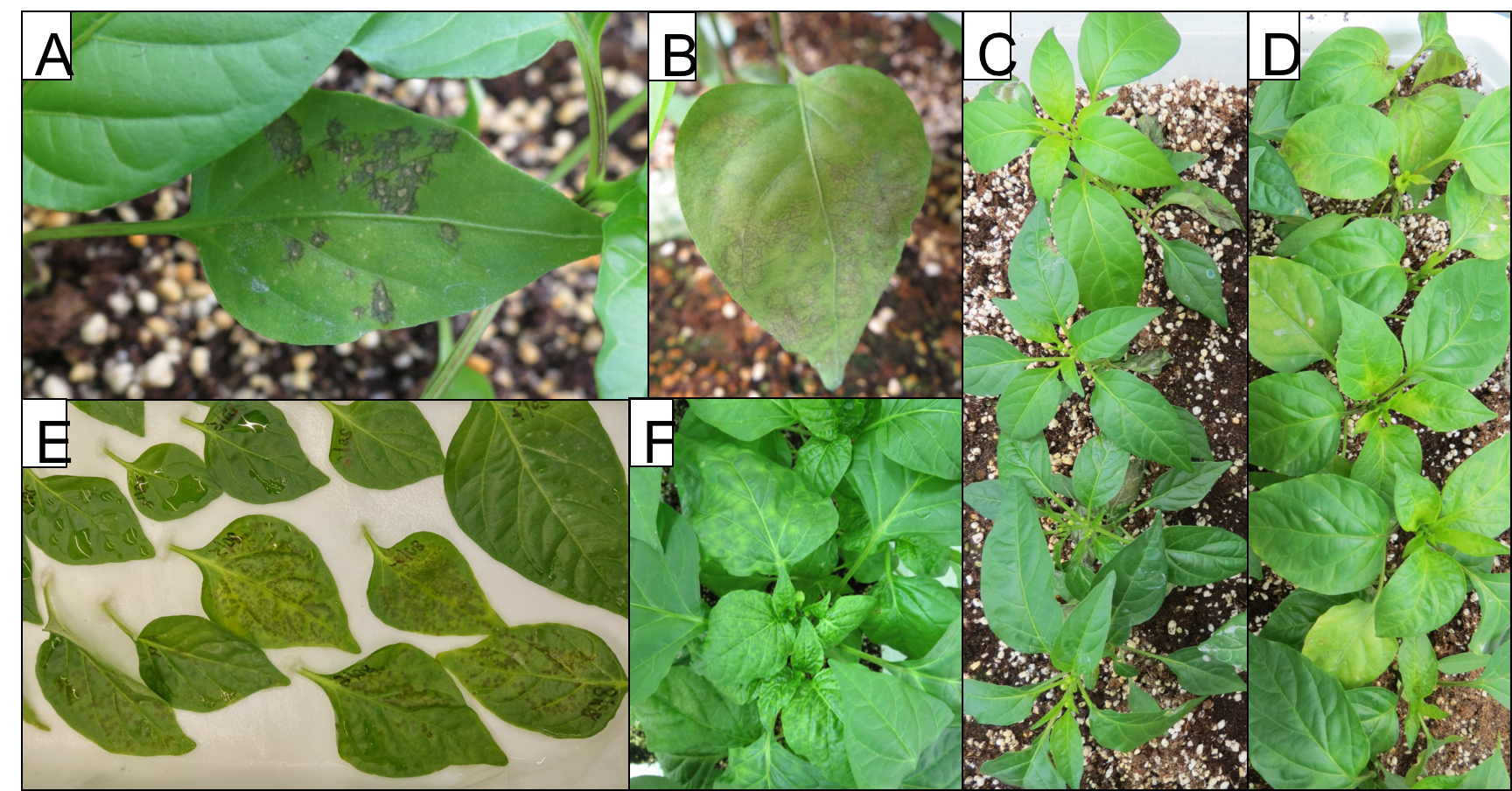

Supplement: S2 Fig — (A) Necrotic local lesion, (B) net like necrosis, (C) leaf abscission, and (D) vein clearing and chlorosis after inoculation with TMV; (E) Necrotic local lesions 3 days after inoculation with PMMoV in detached leaf test; (F) mosaic and diffuse chlorotic spots 6–8 dpi with TSWV. (TIF) [file pone.0237741.s003.tif]

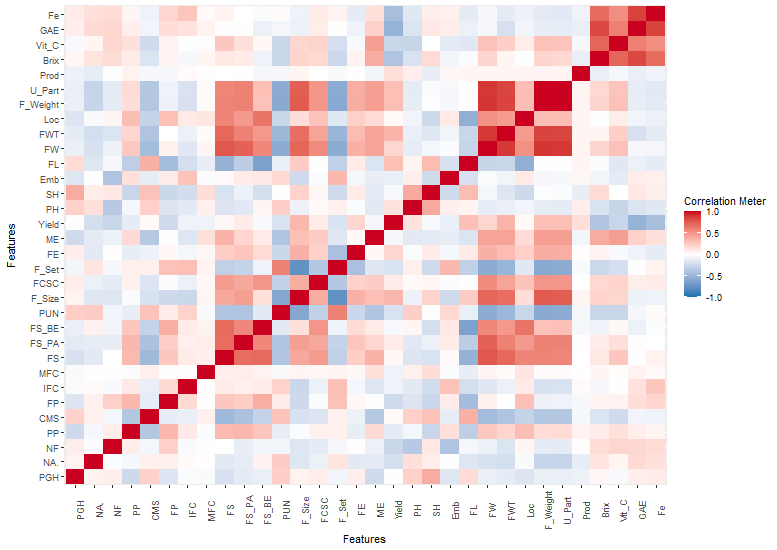

Supplement: S3 Fig — (TIF) [file pone.0237741.s004.tif]
