# Supplementary material for: A step towards Balkan Capsicum annuum L. core collection: Phenotypic and biochemical characterization of 180 accessions for agronomic, fruit quality, and virus resistance traits
Source: PLoS One. 2020 Aug 17;15(8):e0237741. doi: 10.1371/journal.pone.0237741 (PMC7430755; doi:10.1371/journal.pone.0237741)
Supplement: S1 Table — (DOCX) [file pone.0237741.s005.docx]

**S1 Table. Descriptive statistics and analysis of variance (ANOVA) of fruit agro-morphological and productivity traits within varietal groups (VGs)**

|  |  |  |  | **1A. Descriptive Stat** | | | | | |  | **1B. ANOVA** | | | |
| --- | --- | --- | --- | --- | --- | --- | --- | --- | --- | --- | --- | --- | --- | --- |
| **VGs** | **Trait** (Unit) | **N** |  | **Min** | **Mean** | **Max** | **CV (%)** | | **LSD_0.05_** |  | **Accession** | **Year** | **Accession*Year (A*Y) Interaction** | |
| **Pungent** | **Plant Height** (cm) | 49 |  | 41.08 | 81.26 | 114.08 | 14.90 | 9.71 | |  | 15.17*** | 13.22*** | 3.17*** |  |
|  | **Stem height** (cm) | 49 |  | 12.13 | 23.17 | 30.42 | 22.29 | 4.14 | |  | 7.13*** | 180.97*** | 6.05*** |  |
|  | **Embranchment** | 49 |  | 2.25 | 2.72 | 4.54 | 17.93 | 0.39 | |  | 8.86*** | 15.73*** | 6.90*** |  |
|  | **Fruit Length** (cm) | 49 |  | 1.37 | 10.18 | 22.75 | 13.92 | 1.14 | |  | 210.63*** | 0.37 | 5.13*** |  |
|  | **Fruit Width** (cm) | 49 |  | 0.94 | 2.43 | 7.05 | 14.33 | 0.28 | |  | 86.73*** | 149.77*** | 5.39*** |  |
|  | **Fruit Wall Thickness** (mm) | 49 |  | 1.03 | 2.39 | 5.78 | 20.05 | 0.38 | |  | 23.67*** | 100.70*** | 3.26*** |  |
|  | **Locules** | 49 |  | 2.00 | 2.43 | 3.83 | 18.69 | 0.36 | |  | 7.36*** | 12.77*** | 3.13*** |  |
|  | **Fruit Weight** (g) | 49 |  | 1.23 | 23.24 | 92.98 | 30.34 | 5.66 | |  | 68.62*** | 42.40*** | 5.21*** |  |
|  | **Usable Part** (g) | 49 |  | 0.84 | 18.32 | 71.27 | 32.92 | 4.84 | |  | 64.41*** | 26.12*** | 4.48*** |  |
|  | **Productivity** (kg/plant) | 49 |  | 0.22 | 0.51 | 1.26 | 29.10 | 0.17 | |  | 10.43*** | 7.93** | 3.33*** |  |
| **Sweet Green** | **Plant Height** (cm) | 48 |  | 57.75 | 75.12 | 96.33 | 11.87 | 7.15 | |  | 12.02*** | 35.48*** | 5.87*** |  |
|  | **Stem height** (cm) | 48 |  | 14.17 | 22.75 | 31.50 | 21.24 | 3.88 | |  | 8.96*** | 38.18*** | 3.58*** |  |
|  | **Embranchment** | 48 |  | 2.17 | 2.69 | 3.50 | 18.88 | 1.97 | |  | 2.82*** | 0.01 | 1.77** |  |
|  | **Fruit Length** (cm) | 48 |  | 6.38 | 11.14 | 22.67 | 28.80 | 2.57 | |  | 21.43*** | 6.73** | 1.62** |  |
|  | **Fruit Width** (cm) | 48 |  | 1.63 | 4.55 | 7.08 | 12.29 | 0.45 | |  | 88.11*** | 26.09*** | 5.47*** |  |
|  | **Fruit Wall Thickness** (mm) | 48 |  | 1.78 | 3.58 | 5.54 | 19.47 | 0.56 | |  | 19.24*** | 40.12*** | 3.82*** |  |
|  | **Locules** | 48 |  | 2.08 | 2.77 | 3.58 | 19.18 | 0.43 | |  | 8.06*** | 0.61 | 1.71** |  |
|  | **Fruit Weight** (g) | 48 |  | 7.95 | 66.99 | 127.99 | 24.78 | 13.32 | |  | 34.65*** | 0.72 | 3.68*** |  |
|  | **Usable Part** (g) | 48 |  | 5.09 | 56.97 | 113.40 | 26.99 | 12.33 | |  | 32.61*** | 0.73 | 3.95*** |  |
|  | **Productivity** (kg/plant) | 48 |  | 0.31 | 0.61 | 0.95 | 32.38 | 0.23 | |  | 2.37*** | 0.02 | 2.81*** |  |
| **Kapia** | **Plant Height** (cm) | 54 |  | 50.17 | 72.52 | 90.42 | 12.89 | 8.19 | |  | 9.36*** | 9.72** | 7.09*** |  |
|  | **Stem height** (cm) | 54 |  | 10.00 | 23.71 | 31.17 | 20.63 | 4.29 | |  | 6.86*** | 17.56*** | 6.92*** |  |
|  | **Embranchment** | 54 |  | 2.25 | 2.62 | 3.00 | 17.52 | 0.40 | |  | 2.45*** | 31.24*** | 1.72** |  |
|  | **Fruit Length** (cm) | 54 |  | 4.70 | 12.36 | 16.27 | 11.25 | 1.22 | |  | 12.92*** | 12.92*** | 5.21*** |  |
|  | **Fruit Width** (cm) | 54 |  | 1.48 | 4.98 | 8.50 | 22.56 | 0.51 | |  | 12.20*** | 36.76*** | 6.86*** |  |
|  | **Fruit Wall Thickness** (mm) | 54 |  | 1.91 | 3.72 | 6.00 | 17.68 | 0.58 | |  | 13.18*** | 1.45 | 3.45*** |  |
|  | **Locules** | 54 |  | 2.00 | 2.52 | 4.00 | 19.82 | 0.44 | |  | 4.05*** | 29.78*** | 2.01*** |  |
|  | **Fruit Weight** (g) | 54 |  | 15.34 | 92.74 | 180.64 | 17.92 | 14.56 | |  | 46.41*** | 7.77** | 7.96*** |  |
|  | **Usable Part** (g) | 54 |  | 13.20 | 78.22 | 160.58 | 19.55 | 13.40 | |  | 46.86*** | 24.05*** | 8.23*** |  |
|  | **Productivity** (kg/plant) | 54 |  | 0.24 | 0.58 | 1.05 | 35.67 | 0.23 | |  | 3.67*** | 20.18*** | 2.22*** |  |
| **Pumpkin Shape** | **Plant Height** (cm) | 23 |  | 57.92 | 75.77 | 88.75 | 13.81 | 8.43 | |  | 6.75*** | 2.38 | 1.73* |  |
|  | **Stem height** (cm) | 23 |  | 16.33 | 21.78 | 27.50 | 18.34 | 3.81 | |  | 5.16*** | 9.10** | 3.96*** |  |
|  | **Embranchment** | 23 |  | 2.25 | 2.64 | 3.25 | 21.70 | 0.39 | |  | 3.36*** | 6.32*** | 1.75* |  |
|  | **Fruit Length** (cm) | 23 |  | 2.05 | 3.92 | 5.26 | 18.13 | 0.57 | |  | 12.82*** | 94.80*** | 3.43*** |  |
|  | **Fruit Width** (cm) | 23 |  | 4.09 | 7.00 | 8.65 | 11.15 | 0.63 | |  | 24.36*** | 152.12*** | 5.01*** |  |
|  | **Fruit Wall Thickness** (mm) | 23 |  | 4.05 | 5.44 | 6.66 | 15.86 | 0.70 | |  | 4.35*** | 0.74 | 4.18*** |  |
|  | **Locules** | 23 |  | 2.67 | 3.07 | 3.58 | 17.41 | 0.43 | |  | 2.41*** | 1.31 | 2.47*** |  |
|  | **Fruit Weight** (g) | 23 |  | 35.88 | 111.61 | 151.82 | 22.57 | 20.31 | |  | 16.67*** | 24.11*** | 2.59*** |  |
|  | **Usable Part** (g) | 23 |  | 28.38 | 95.99 | 131.57 | 23.94 | 18.52 | |  | 16.65*** | 39.06*** | 2.91*** |  |
|  | **Productivity** (kg/plant) | 23 |  | 0.25 | 0.51 | 0.74 | 37.64 | 0.23 | |  | 2.27** | 2.07 | 1.53 |  |
| **Paprika** | **Plant Height** (cm) | 6 |  | 32.50 | 64.44 | 91.58 | 11.12 | 5.86 | |  | 118.16*** | 31.29*** | 8.33*** |  |
|  | **Stem height** (cm) | 6 |  | 18.08 | 25.57 | 29.50 | 14.40 | 3.01 | |  | 18.49*** | 10.04** | 10.44*** |  |
|  | **Embranchment** | 6 |  | 1.17 | 2.40 | 3.08 | 23.20 | 0.46 | |  | 22.13*** | 5.41* | 4.98*** |  |
|  | **Fruit Length** (cm) | 6 |  | 6.73 | 9.06 | 12.61 | 12.38 | 0.92 | |  | 39.60*** | 0.18 | 4.47** |  |
|  | **Fruit Width** (cm) | 6 |  | 2.08 | 2.66 | 3.29 | 12.26 | 0.27 | |  | 29.26*** | 14.39*** | 7.64*** |  |
|  | **Fruit Wall Thickness** (mm) | 6 |  | 1.03 | 1.37 | 1.80 | 30.29 | 0.34 | |  | 7.51*** | 18.98*** | 1.86*** |  |
|  | **Locules** | 6 |  | 2.33 | 2.92 | 3.92 | 18.97 | 0.45 | |  | 12.31*** | 14.70*** | 3.81** |  |
|  | **Fruit Weight** (g) | 6 |  | 9.78 | 16.29 | 23.58 | 20.77 | 2.77 | |  | 36.00*** | 17.03*** | 5.75*** |  |
|  | **Usable Part** (g) | 6 |  | 7.45 | 13.55 | 19.89 | 22.13 | 2.46 | |  | 36.46*** | 29.94*** | 8.52*** |  |
|  | **Productivity** (kg/plant) | 6 |  | 0.11 | 0.18 | 0.24 | 33.13 | 0.07 | |  | 5.11** | 6.51* | 0.94 |  |

Where, SD: Standard Deviation; CV: Coefficient of Variation; LSD: Least Significant Differences. * ** *** showed differences at 0.05, 0.01 and 0.001 significance level, respectively.
