# Supplementary material for: A step towards Balkan Capsicum annuum L. core collection: Phenotypic and biochemical characterization of 180 accessions for agronomic, fruit quality, and virus resistance traits
Source: PLoS One. 2020 Aug 17;15(8):e0237741. doi: 10.1371/journal.pone.0237741 (PMC7430755; doi:10.1371/journal.pone.0237741)
Supplement: S2 Table — (DOCX) [file pone.0237741.s006.docx]

**S2 Table. Descriptive statistics and analysis of variance (ANOVA) of fruit agro-morphological and productivity traits within varietal groups (VGs) evaluated during 2018**

|  |  |  |  | **1A. Descriptive Stat** | | | | | |  | **1B. ANOVA** |
| --- | --- | --- | --- | --- | --- | --- | --- | --- | --- | --- | --- |
| **VGs** | **Trait** (Unit) | **N** |  | **Min** | **Mean** | **Max** | **CV (%)** | | **LSD_0.05_** |  | **Accession** |
| **Pungent** | **Plant Height** (cm) | 48 |  | 31.67 | 83.30 | 123.33 | 13.89 | 13.02 | |  | 11.64*** |
|  | **Stem height** (cm) | 48 |  | 15.00 | 26.12 | 35.50 | 18.45 | 5.42 | |  | 9.31*** |
|  | **Embranchment** | 48 |  | 2.00 | 2.67 | 5.92 | 20.65 | 0.62 | |  | 11.54*** |
|  | **Fruit Length** (cm) | 48 |  | 1.47 | 10.25 | 22.83 | 15.21 | 1.76 | |  | 89.61*** |
|  | **Fruit Width** (cm) | 48 |  | 1.02 | 2.21 | 3.95 | 14.09 | 0.35 | |  | 36.89*** |
|  | **Fruit Wall Thickness** (mm) | 48 |  | 0.95 | 2.16 | 3.55 | 20.28 | 0.49 | |  | 9.25*** |
|  | **Locules** | 48 |  | 2.00 | 2.35 | 3.33 | 18.97 | 0.50 | |  | 2.76*** |
|  | **Fruit Weight** (g) | 48 |  | 1.32 | 20.57 | 55.20 | 31.12 | 7.20 | |  | 31.11*** |
|  | **Usable Part** (g) | 48 |  | 0.88 | 16.44 | 47.12 | 32.90 | 6.09 | |  | 30.96*** |
|  | **Productivity** (kg/plant) | 48 |  | 0.17 | 0.49 | 1.39 | 30.68 | 0.24 | |  | 7.28*** |
| **Sweet Green** | **Plant Height** (cm) | 48 |  | 60.00 | 77.33 | 100.00 | 11.25 | 9.90 | |  | 8.31*** |
|  | **Stem height** (cm) | 48 |  | 13.33 | 23.99 | 34.67 | 21.01 | 5.73 | |  | 7.41*** |
|  | **Embranchment** | 48 |  | 2.17 | 2.69 | 3.33 | 19.57 | 0.60 | |  | 2.33*** |
|  | **Fruit Length** (cm) | 48 |  | 6.02 | 10.79 | 21.00 | 13.44 | 1.65 | |  | 45.57*** |
|  | **Fruit Width** (cm) | 48 |  | 1.65 | 4.43 | 7.07 | 12.42 | 0.63 | |  | 44.67*** |
|  | **Fruit Wall Thickness** (mm) | 48 |  | 1.65 | 3.40 | 5.60 | 20.61 | 0.76 | |  | 12.37*** |
|  | **Locules** | 48 |  | 2.00 | 2.79 | 3.83 | 18.63 | 0.59 | |  | 5.33*** |
|  | **Fruit Weight** (g) | 48 |  | 9.22 | 66.41 | 125.95 | 24.44 | 18.46 | |  | 17.33*** |
|  | **Usable Part** (g) | 48 |  | 5.90 | 56.42 | 110.03 | 26.72 | 17.15 | |  | 16.10*** |
|  | **Productivity** (kg/plant) | 48 |  | 0.30 | 0.61 | 0.99 | 36.00 | 1.99 | |  | 1.74** |
| **Kapia** | **Plant Height** (cm) | 53 |  | 51.67 | 73.65 | 106.67 | 12.78 | 10.65 | |  | 9.02*** |
|  | **Stem height** (cm) | 53 |  | 11.00 | 22.99 | 38.00 | 21.82 | 5.68 | |  | 9.39*** |
|  | **Embranchment** | 53 |  | 2.00 | 2.52 | 3.17 | 17.98 | 0.51 | |  | 3.05*** |
|  | **Fruit Length** (cm) | 53 |  | 9.73 | 12.27 | 18.87 | 11.29 | 1.57 | |  | 9.47*** |
|  | **Fruit Width** (cm) | 53 |  | 1.55 | 4.65 | 6.82 | 13.25 | 0.70 | |  | 4.65*** |
|  | **Fruit Wall Thickness** (mm) | 52 |  | 1.89 | 3.68 | 5.46 | 19.28 | 0.80 | |  | 7.10*** |
|  | **Locules** | 53 |  | 2.00 | 2.62 | 3.33 | 19.87 | 0.59 | |  | 2.76*** |
|  | **Fruit Weight** (g) | 53 |  | 16.97 | 94.26 | 188.58 | 17.19 | 18.33 | |  | 30.44*** |
|  | **Usable Part** (g) | 53 |  | 15.13 | 80.89 | 166.92 | 18.14 | 16.60 | |  | 32.34*** |
|  | **Productivity** (kg/plant) | 53 |  | 0.24 | 0.63 | 1.38 | 39.22 | 0.40 | |  | 3.10*** |
| **Pumpkin Shape** | **Plant Height** (cm) | 23 |  | 59.17 | 76.70 | 91.67 | 13.11 | 11.51 | |  | 4.49*** |
|  | **Stem height** (cm) | 23 |  | 12.33 | 20.92 | 30.00 | 23.98 | 5.74 | |  | 4.28*** |
|  | **Embranchment** | 23 |  | 2.00 | 2.57 | 3.50 | 20.96 | 0.62 | |  | 3.17*** |
|  | **Fruit Length** (cm) | 23 |  | 2.00 | 3.52 | 5.25 | 20.92 | 0.84 | |  | 8.43*** |
|  | **Fruit Width** (cm) | 23 |  | 3.58 | 6.44 | 8.65 | 13.74 | 1.01 | |  | 13.58*** |
|  | **Fruit Wall Thickness** (mm) | 23 |  | 4.09 | 5.48 | 7.44 | 16.58 | 1.04 | |  | 4.84*** |
|  | **Locules** | 23 |  | 2.33 | 3.03 | 3.50 | 18.51 | 0.64 | |  | 1.48 |
|  | **Fruit Weight** (g) | 23 |  | 34.85 | 118.91 | 172.20 | 24.08 | 32.76 | |  | 9.69*** |
|  | **Usable Part** (g) | 23 |  | 27.18 | 104.47 | 151.62 | 25.26 | 30.20 | |  | 9.78*** |
|  | **Productivity** (kg/plant) | 23 |  | 0.13 | 0.52 | 0.81 | 42.98 | 2.02 | |  | 1.39 |
| **Paprika** | **Plant Height** (cm) | 6 |  | 30.00 | 69.17 | 98.33 | 8.70 | 7.16 | |  | 125.28*** |
|  | **Stem height** (cm) | 6 |  | 18.33 | 26.94 | 36.67 | 14.70 | 4.71 | |  | 16.75*** |
|  | **Embranchment** | 6 |  | 2.00 | 2.56 | 3.00 | 25.29 | 0.77 | |  | 3.62** |
|  | **Fruit Length** (cm) | 6 |  | 7.45 | 9.11 | 13.62 | 12.83 | 1.39 | |  | 23.81*** |
|  | **Fruit Width** (cm) | 6 |  | 2.17 | 2.81 | 3.28 | 12.03 | 0.40 | |  | 9.38*** |
|  | **Fruit Wall Thickness** (mm) | 6 |  | 0.92 | 1.16 | 1.50 | 32.24 | 0.44 | |  | 1.16*** |
|  | **Locules** | 6 |  | 4.67 | 3.17 | 2.33 | 17.86 | 0.67 | |  | 12.71*** |
|  | **Fruit Weight** (g) | 6 |  | 8.70 | 14.64 | 21.68 | 21.52 | 3.75 | |  | 17.30*** |
|  | **Usable Part** (g) | 6 |  | 5.80 | 11.62 | 18.53 | 23.77 | 3.28 | |  | 18.92*** |
|  | **Productivity** (kg/plant) | 6 |  | 0.08 | 0.15 | 0.21 | 40.04 | 2.23 | |  | 2.83 |

Where, SD: Standard Deviation; CV: Coefficient of Variation; LSD: Least Significant Differences. * ** *** showed differences at 0.05, 0.01 and 0.001 significance level, respectively.
