# Supplementary material for: A step towards Balkan Capsicum annuum L. core collection: Phenotypic and biochemical characterization of 180 accessions for agronomic, fruit quality, and virus resistance traits
Source: PLoS One. 2020 Aug 17;15(8):e0237741. doi: 10.1371/journal.pone.0237741 (PMC7430755; doi:10.1371/journal.pone.0237741)
Supplement: S3 Table — (DOCX) [file pone.0237741.s007.docx]

**S3 Table. Descriptive statistics and analysis of variance (ANOVA) of fruit agro-morphological and productivity traits within varietal groups (VGs) evaluated during 2019**

|  |  |  |  | **1A. Descriptive Stat** | | | | | |  | **1B. ANOVA** |
| --- | --- | --- | --- | --- | --- | --- | --- | --- | --- | --- | --- |
| **VGs** | **Trait** (Unit) | **N** |  | **Min** | **Mean** | **Max** | **CV (%)** | | **LSD_0.05_** |  | **Accession** |
| **Pungent** | **Plant Height** (cm) | 49 |  | 45.83 | 79.26 | 104.83 | 15.79 | 14.09 | |  | 7.35*** |
|  | **Stem height** (cm) | 49 |  | 7.33 | 20.27 | 30.83 | 26.58 | 6.06 | |  | 4.70*** |
|  | **Embranchment** | 49 |  | 2.33 | 2.78 | 3.17 | 14.99 | 0.47 | |  | 1.71** |
|  | **Fruit Length** (cm) | 49 |  | 1.27 | 10.12 | 22.67 | 12.38 | 1.41 | |  | 140.22*** |
|  | **Fruit Width** (cm) | 49 |  | 0.87 | 2.65 | 7.05 | 14.31 | 0.43 | |  | 53.45*** |
|  | **Fruit Wall Thickness** (mm) | 49 |  | 1.12 | 2.61 | 5.78 | 19.83 | 0.58 | |  | 16.50*** |
|  | **Locules** | 49 |  | 2.00 | 2.50 | 0.52 | 18.43 | 3.83 | |  | 7.58*** |
|  | **Fruit Weight** (g) | 49 |  | 1.07 | 25.86 | 92.98 | 29.32 | 8.53 | |  | 42.05*** |
|  | **Usable Part** (g) | 49 |  | 0.53 | 20.16 | 71.27 | 32.50 | 7.37 | |  | 37.61*** |
|  | **Productivity** (kg/plant) | 49 |  | 0.16 | 0.53 | 1.12 | 27.79 | 0.24 | |  | 6.55*** |
| **Sweet Green** | **Plant Height** (cm) | 48 |  | 43.83 | 72.91 | 104.33 | 12.57 | 10.42 | |  | 9.45*** |
|  | **Stem height** (cm) | 48 |  | 13.17 | 21.50 | 31.50 | 21.29 | 5.21 | |  | 4.99*** |
|  | **Embranchment** | 48 |  | 2.00 | 2.69 | 3.83 | 18.26 | 0.56 | |  | 2.23*** |
|  | **Fruit Length** (cm) | 48 |  | 6.12 | 11.49 | 24.33 | 37.24 | 4.87 | |  | 7.72*** |
|  | **Fruit Width** (cm) | 48 |  | 1.62 | 4.67 | 8.55 | 12.05 | 0.64 | |  | 49.71*** |
|  | **Fruit Wall Thickness** (mm) | 48 |  | 1.92 | 3.76 | 6.47 | 18.22 | 0.78 | |  | 10.94*** |
|  | **Locules** | 48 |  | 2.00 | 2.76 | 4.00 | 19.79 | 0.62 | |  | 4.45*** |
|  | **Fruit Weight** (g) | 48 |  | 6.68 | 67.59 | 142.08 | 24.88 | 19.12 | |  | 21.21*** |
|  | **Usable Part** (g) | 48 |  | 4.28 | 57.51 | 126.82 | 26.96 | 17.64 | |  | 20.71*** |
|  | **Productivity** (kg/plant) | 48 |  | 0.26 | 0.61 | 1.16 | 27.75 | 0.28 | |  | 4.12*** |
| **Kapia** | **Plant Height** (cm) | 54 |  | 46.67 | 71.40 | 110.00 | 12.67 | 10.71 | |  | 7.84*** |
|  | **Stem height** (cm) | 54 |  | 10.00 | 24.43 | 33.33 | 19.28 | 5.57 | |  | 4.26*** |
|  | **Embranchment** | 54 |  | 2.33 | 2.73 | 3.17 | 16.75 | 0.54 | |  | 1.25 |
|  | **Fruit Length** (cm) | 54 |  | 4.70 | 12.44 | 16.30 | 11.14 | 1.64 | |  | 8.87*** |
|  | **Fruit Width** (cm) | 54 |  | 1.40 | 5.32 | 8.50 | 9.90 | 0.62 | |  | 30.76*** |
|  | **Fruit Wall Thickness** (mm) | 54 |  | 1.92 | 3.76 | 6.00 | 16.07 | 0.72 | |  | 10.02*** |
|  | **Locules** | 54 |  | 2.00 | 2.42 | 4.00 | 19.50 | 0.56 | |  | 3.47*** |
|  | **Fruit Weight** (g) | 54 |  | 13.72 | 91.21 | 195.17 | 18.54 | 20.01 | |  | 24.93*** |
|  | **Usable Part** (g) | 54 |  | 11.27 | 75.56 | 170.75 | 20.92 | 18.70 | |  | 24.08*** |
|  | **Productivity** (kg/plant) | 54 |  | 0.24 | 0.53 | 0.94 | 28.28 | 0.24 | |  | 2.82*** |
| **Pumpkin Shape** | **Plant Height** (cm) | 23 |  | 52.50 | 74.84 | 89.50 | 14.32 | 12.31 | |  | 4.11*** |
|  | **Stem height** (cm) | 23 |  | 15.00 | 22.65 | 29.17 | 19.82 | 5.16 | |  | 4.31*** |
|  | **Embranchment** | 23 |  | 2.33 | 2.72 | 3.00 | 16.05 | 0.50 | |  | 1.54 |
|  | **Fruit Length** (cm) | 23 |  | 2.10 | 4.33 | 5.27 | 15.86 | 0.79 | |  | 8.65*** |
|  | **Fruit Width** (cm) | 23 |  | 4.60 | 7.57 | 9.08 | 8.80 | 0.77 | |  | 17.73*** |
|  | **Fruit Wall Thickness** (mm) | 23 |  | 3.72 | 5.40 | 6.35 | 15.37 | 0.95 | |  | 3.23*** |
|  | **Locules** | 23 |  | 2.50 | 3.10 | 4.17 | 16.26 | 0.58 | |  | 3.50*** |
|  | **Fruit Weight** (g) | 23 |  | 36.90 | 104.26 | 138.25 | 20.84 | 24.97 | |  | 8.81*** |
|  | **Usable Part** (g) | 23 |  | 29.58 | 87.47 | 117.68 | 22.08 | 22.20 | |  | 8.98*** |
|  | **Productivity** (kg/plant) | 23 |  | 0.25 | 0.51 | 0.74 | 39.64 | 0.24 | |  | 2.02** |
| **Paprika** | **Plant Height** (cm) | 6 |  | 35.00 | 59.72 | 84.83 | 12.38 | 8.79 | |  | 35.75*** |
|  | **Stem height** (cm) | 6 |  | 17.83 | 24.19 | 29.67 | 13.00 | 3.74 | |  | 13.10*** |
|  | **Embranchment** | 6 |  | 0.33 | 2.25 | 3.17 | 21.00 | 0.56 | |  | 30.97*** |
|  | **Fruit Length** (cm) | 6 |  | 6.00 | 9.00 | 11.60 | 11.40 | 1.22 | |  | 21.73*** |
|  | **Fruit Width** (cm) | 6 |  | 1.75 | 2.51 | 3.42 | 12.79 | 0.38 | |  | 27.64*** |
|  | **Fruit Wall Thickness** (mm) | 6 |  | 1.13 | 1.58 | 2.10 | 28.63 | 0.54 | |  | 6.68*** |
|  | **Locules** | 6 |  | 2.17 | 2.67 | 3.17 | 19.37 | 0.61 | |  | 3.25* |
|  | **Fruit Weight** (g) | 6 |  | 8.75 | 17.93 | 25.47 | 19.28 | 4.11 | |  | 25.59*** |
|  | **Usable Part** (g) | 6 |  | 6.37 | 15.49 | 22.58 | 19.97 | 3.68 | |  | 27.23*** |
|  | **Productivity** (kg/plant) | 6 |  | 0.13 | 0.20 | 0.29 | 27.14 | 0.10 | |  | 3.44* |

Where, SD: Standard Deviation; CV: Coefficient of Variation; LSD: Least Significant Differences. * ** *** showed differences at 0.05, 0.01 and 0.001 significance level, respectively.
